# Bacteriocyte plasticity in pea aphids facing amino acid stress or starvation during development

**DOI:** 10.3389/fphys.2022.982920

**Published:** 2022-11-10

**Authors:** Mélanie Ribeiro Lopes, Karen Gaget, François Renoz, Gabrielle Duport, Séverine Balmand, Hubert Charles, Patrick Callaerts, Federica Calevro

**Affiliations:** ^1^ Université de Lyon, INSA Lyon, INRAE, BF2I, UMR 203, Villeurbanne, France; ^2^ Université de Lyon, INRAE, INSA Lyon, BF2I, UMR 203, Villeurbanne, France; ^3^ UCLouvain, Biodiversity Research Centre, Earth and Life Institute, Louvain-la-Neuve, Belgium; ^4^ KU Leuven, Laboratory of Behavioral and Developmental Genetics, Department of Human Genetics, Leuven, Belgium

**Keywords:** aphids, *Acyrthosiphon pisum*, symbiosis, bacteriocytes, amino acid stress, starvation

## Abstract

An important contributing factor to the evolutionary success of insects is nutritional association with microbial symbionts, which provide the host insects with nutrients lacking in their unbalanced diets. These symbionts are often compartmentalized in specialized cells of the host, the bacteriocytes. Even though bacteriocytes were first described more than a century ago, few studies have explored their dynamics throughout the insect life cycle and in response to environmental stressors. Here, we use the *Buchnera aphidicola*/pea aphid symbiotic system to study how bacteriocytes are regulated in response to nutritional stress throughout aphid development. Using artificial diets, we analyzed the effects of depletion or excess of phenylalanine or leucine, two amino acids essential for aphid growth and whose biosynthetic pathways are shared between the host and the symbiont. Bacteriocytes responded dynamically to those treatments, while other tissues showed no obvious morphological change. Amino acid depletion resulted in an increase in bacteriocyte numbers, with the extent of the increase depending on the amino acid, while excess either caused a decrease (for leucine) or an increase (for phenylalanine). Only a limited impact on survival and fecundity was observed, suggesting that the adjustment in bacteriocyte (and symbiont) numbers is sufficient to withstand these nutritional challenges. We also studied the impact of more extreme conditions by exposing aphids to a 24 h starvation period at the beginning of nymphal development. This led to a dramatic drop in aphid survival and fecundity and a significant developmental delay. Again, bacteriocytes responded dynamically, with a considerable decrease in number and size, correlated with a decrease in the number of symbionts, which were prematurely degraded by the lysosomal system. This study shows how bacteriocyte dynamics is integrated in the physiology of insects and highlights the high plasticity of these cells.

## Introduction

Insects constitute nearly 90% of animal species known to date and are found in a wide variety of habitats (Douglas 2014). An important contributing factor to their evolutionary success is the association with obligatory symbiotic bacteria, which enables the insect hosts to thrive on specialized ecological niches by providing essential nutrients that are in short supply in their unbalanced diets (Wernegreen 2012; Hansen and Moran 2014). In those systems, the genomes of the eukaryotic host and the prokaryotic partner are complementary for several biosynthetic pathways that control the production of the deficient nutrients, resulting in a highly integrated metabolism (Douglas 2014; Hansen and Moran 2014; Ankrah et al., 2017). Many insect hosts have evolved specialized cells, called bacteriocytes, that can be organized in multicellular bacteriomes, whose function is to house and regulate symbiont populations (Buchner 1965; Douglas 1989; Matsuura et al., 2015; Sudakaran et al., 2017). Obligatory symbiotic bacteria are maintained within those symbiont-bearing cells through vertical transmission across host generations (Koga et al., 2012; Luan et al., 2018; Kuechler et al., 2019; Michalik et al., 2019). While the presence of bacteriocytes is now well documented in at least six different insect orders (i.e. Dictyoptera, Coleoptera, Diptera, Hymenoptera, Hemiptera, and Phthiraptera (Douglas 1989; Matsuura et al., 2015; Szklarzewicz and Michalik 2017), only a few studies have explored the dynamics of both the bacteriocytes and the symbionts, with most of them focusing on specific life stages without considering the entire insect life cycle (Stoll et al., 2009; Stoll et al., 2010; Vigneron et al., 2014).

The most detailed characterization of the growth dynamics of bacteriocytes and associated symbiotic bacteria was carried out in the pea aphid *Acyrthosiphon pisum/Buchnera aphidicola* symbiosis, which has emerged in recent years as a powerful model for the study of bacteriocytes (Simonet et al., 2016a; Simonet et al., 2018; Pers and Hansen 2021). In this system, bacteriocyte analyses are facilitated by their large size (*e.g.* diameter that can exceed 100 µm in adults) and their organization in clusters of weakly adherent cells that can readily be isolated (Simonet et al., 2018) compared to insects where they form a true bacteriome (Hosokawa et al., 2010; Vigneron et al., 2014; Kuechler et al., 2019). In addition, a flow cytometry-based approach has recently been developed for the quantification of the absolute number of *B. aphidicola* (Simonet et al., 2016a). This method is more accurate than qPCR-based approaches that only provide information on the relative copy numbers of a *Buchnera* gene versus an aphid gene. Flow cytometry is also preferable to qPCR in this symbiotic system due to the polyploidy of both *B. aphidicola* and bacteriocytes, whose genome copy number can moreover vary during insect development (Komaki and Ishikawa 2000; Nozaki and Shigenobu 2022). Studies on the pea aphid have shown that bacteriocytes, coordinately with the *B. aphidicola* endosymbiont population, grow considerably during nymphal development when high nutritional complementation is required for the development of the insect and that of its embryos (Bermingham and Wilkinson 2009). Once adulthood has been reached and the nymph-laying period has started, the maintenance of a very dense symbiont population becomes excessively costly for the insect. At that moment, a second phase starts, with a gradual reorganization of bacteriocyte structure as the aphid ages (*e.g.* loss of cohesion, reduction in number and size, decrease in the number of endosymbionts). During this phase, bacteriocytes are eliminated by a non-apoptotic cell death process, initiated by a massive vacuolization of the cytoplasm originating from the endoplasmic reticulum, and symbionts are degraded by the lysosomal system (Simonet et al., 2018).

A recent study (Colella et al., 2018) of the *A. pisum/B. aphidicola* symbiotic system has revealed that not the gut, but the bacteriocytes, are highly responsive when the insects are submitted to a total depletion of phenylalanine (Phe) and tyrosine (Tyr), two amino acids jointly produced by the two symbiotic partners and essential for their development and growth. The study showed that bacteriocytes undergo 1) extensive transcriptional reprogramming, with up-regulation of division and cell growth-associated genes, and 2) an increase in their number following the Phe/Tyr depletion. This is particularly interesting as aphids naturally experience many nutritional challenges (either deficiency or excess) as their host plants change and grow, or as their location varies. Indeed, as other hemipteran insects, aphids feed exclusively on plant phloem sap, a diet rich in carbohydrates (*e.g.* sucrose) but poor in essential amino acids with a composition that is highly variable depending on the species and age of the plant, and the environment (Sharkey and Pate 1976; Smith and Milburn 1980; Hayashi and Chino 1990; Winter et al., 1992; Corbesier et al., 2001; Douglas 2003; Gholami et al., 2004; Douglas 2006; Gattolin et al., 2008). Aphids are also known to drop off their host plant in the presence of predators or in case of high population density (Dill et al., 1990; Bailey et al., 1995; Mann et al., 1995; Gish et al., 2010; Roitberg and Myers 2012), which regularly confronts them with short periods of starvation. In this context, changes in the bacteriocytes could represent an adaptive strategy allowing the insect host to cope with dietary and/or environmental challenges. However, many questions remain unanswered. Do bacteriocytes respond similarly to a depletion of other amino acids than Phe and Tyr? Can we expect an amino acid excess to have the opposite effect and lead to a decrease in the number of bacteriocytes? How do bacteriocytes react to more extreme nutritional challenges such as starvation? Is the number of symbionts directly correlated with the bacteriocyte dynamics? How are these changes regulated with regard to insect development and bacteriocyte proliferation/cell death?

To address these questions, we first used artificial diets to test the impact of both depletion and excess of single specific amino acids on aphid physiology and bacteriocyte dynamics, using the pea aphid as a model system. We chose to focus on Phe and Leucine (Leu) because 1) they are known to be important for the growth of aphids (Viñuelas et al., 2011) and that of their embryos (Douglas 1996; Wilkinson and Ishikawa 1999; Bermingham and Wilkinson 2009; Bermingham and Wilkinson 2010), 2) their concentration in the plant phloem sap can be very limited and changing throughout the day (Karley et al., 2002; Douglas 2006; Gattolin et al., 2008) and 3) their respective biosynthetic pathways are well annotated (The International Aphid Genomics Consortium 2010; Wilson et al., 2010), which allow interpretation of the biochemical basis of the phenotypic effects caused by changes in their concentration. We also studied the impact of more extreme conditions by starving aphids for 24 h. Our study shows that bacteriocytes react differently to distinct nutritional challenges. Depletion of single amino acids systematically resulted in an increase in bacteriocyte numbers, with the size of the response dependent on the amino acids, while excess either caused a decrease or an increase. Overall, these stresses had limited impact on aphid survival and fecundity, suggesting that the changes in bacteriocyte number constitute an adequate response to nutritional stress. By contrast, an early exposure to a 24 h starvation period led to a dramatic drop in survival and fecundity rates as well as a significant developmental delay. We simultaneously observed a significant decrease in bacteriocyte number and size, highly correlated to a decrease in the number of symbionts, which are prematurely degraded by the lysosomal system. This study suggests that bacteriocytes are major players in the response of a symbiotic insect to nutritional stress.

## Materials and methods

### Aphid rearing and sampling

The pea aphids *A. pisum* (Harris) used in this study were obtained from a long-established parthenogenetic clone (LL01), which is mono-symbiotic and contains only the primary endosymbiont *B. aphidicola*. The absence of secondary symbionts that regularly occurr in pea aphids (i.e. *Hamiltonella defensa*, *cd. Fukatsuia symbiotica* (previously known as Pea Aphid X-type Symbiont or PAXS), *Regiella insecticola*, *Rickettsia* sp, *Rickettsiella* sp, *Serratia symbiotica* and *Spiroplasma* sp.) was verified by means of a previously described PCR-based diagnostic (Peccoud et al., 2014). Aphids were kept on young broad bean plants (*Vicia faba,* L. cv. Aquadulce), at 21°C, with a photoperiod of 16 h light—8 h dark allowing us to maintain them as strictly parthenogenetic matrilines. To produce synchronized apterous nymphs, winged adults were allowed to reproduce on seedlings for 24 h, then removed as previously described (Sapountzis et al., 2014). The resulting synchronized first instar nymphs (<1 day old) were then transferred either directly to new plants, placed on artificial media or subjected to starvation treatments depending on the experiment (Supplementary Figure S1). Aphid development was monitored by inspecting their molts. Aphids were considered third or fourth instar nymphs when they had completed the second or third molt, respectively. Under our laboratory conditions, this corresponds to day 4 or day 6, respectively, both for aphids reared on plants or on artificial diets (Simonet et al., 2016b; Colella et al., 2018). Aphids were sampled at different developmental stages: third instar (N3; 5 days old) and fourth instar (N4; 7 days old) nymphs; and adults A8 (8 days old), A9 (9 days old), A12 (12 days old), A14 (14 days old), A15 (15 days old), A16 (16 days old), A19 (19 days old) and A23 (23 days old). All nymphs and adults were randomly collected from the synchronized source population. Unless stated otherwise, all experiments were conducted independently, starting from distinct synchronized stocks.

### Artificial diets and starvation treatments

For the experiment testing the effect of single amino acid depletion or excess, first instar nymphs were collected and placed on artificial media in *ad hoc* feeding chambers, as originally described by Febvay *et al.* (1988). Five different artificial media were used in this experiment. The control AP3 diet was originally defined by combining data on the amino acid composition of phloem sap of leguminous plants and aphid hemolymph (Febvay et al., 1988; Calatayud 2000). The Leu0 and Phe0 diets corresponded to an AP3 diet without Leu (0 mm) or Phe (0 mm), respectively. The Leu80 and Phe60 diets were the same as AP3 but with amounts of Leu or Phe equivalent to four or three times their concentration in AP3 (80 mm and 60 mm, respectively). Following the preparation of the artificial diets, the actual concentrations of the amino acids were verified by HPLC analysis following the protocol established by Febvay *et al.* (1999). For the starvation experiment, aphids were placed in different conditions: Petri dishes (60 mm in diameter, five individuals per dish) with or without a moist filter paper to prevent desiccation; feeding chambers (60 mm in diameter, five individuals per feeding chamber) containing water; feeding chambers that contained sugary water (20% sucrose) with or without the vitamins from the AP3 diet (“Sugar + Vitamins” and “Sugar” diets, respectively) (Supplementary Figure S1B; Supplementary Table S1). The refeeding experiments consisted of 24 h starvation in Petri dishes fitted with a moist filter, followed by the transfer of surviving aphids (between 1 and 2 days old) to young seedlings (Supplementary Figure S1C). We chose to use plants instead of artificial diets for the refeeding experiments for two main reasons: 1) to place aphids in physiological conditions that are closest to those naturally encountered in the field and 2) because we observed that aphids do not readily feed on artificial diets if they have not been reared on them since birth.

### Life history trait measurements

First instar nymphs (40–60 for each condition) were monitored daily for survival and the presence of possible phenotypic and/or behavioral effects. An independent experiment was performed to assess fecundity using ten additional aphids per condition that were isolated at day 8, the time point of transition to adulthood in aphids reared on plants. The number of newly deposited nymphs was counted daily. For the refeeding experiments, 10 aphids per developmental stage and condition were collected and their weight scored individually with a Mettler AE163 analytical microbalance (Mettler Toledo, Columbus, OH, United States).

### Counting and size determination of aphid bacteriocytes

Bacteriocytes were isolated from the abdomen of each individual aphid in ice-cold buffer A (0.025 M KCl, 0.01 M MgCl_2_, 0.25 M Sucrose and 0.035 M Tris-HCl, pH 7.5) and counted at 25–40X magnification with an MDG-17 stereomicroscope (Leica, Wild Heerbrugg AG, Switzerland), following the protocol previously described (Ribeiro Lopes et al., 2021). The bacteriocytes of 10 aphids were analyzed per developmental stage and condition. Size determination was done on unstained living bacteriocytes that were collected with a Pasteur pipette attached to a peristaltic pump MINIPULS 3 (Gilson, Middleton, WI, United States) and mounted on glass slides, using spacers to prevent crushing, following the procedure we recently developed (Simonet et al., 2016a, 2018). Bright-field images were acquired with an Olympus IX81 microscope (Olympus Corporation, Tokyo, Japan) at ×40 magnification and pictures were taken using an Olympus cooled color camera XC50 (Olympus Corporation) and CellSens software (Olympus Corporation). Since aphid bacteriocytes appear as irregular spherical cells, pictures for individual bacteriocytes were taken at the point where the cell diameter was widest. Because bacteriocytes are often present as clusters of overlapping cells, which precludes the use of segmentation tools to automatically and efficiently measure cell size, bacteriocyte areas were then individually measured using the ImageJ software (https://imagej.nih.gov/ij/). The freehand selection tool of the software was used to delineate the perimeter of each individual bacteriocyte and the pixels of each selection were then converted to the corresponding area in µm^2^.

### Determination of bacterial numbers by flow cytometry

For each developmental stage and condition, symbiotic bacteria from ten whole aphids were purified as previously described (Simonet et al., 2016a). Aphids were gently crushed with a Potter homogenizer in 3 ml of ice-cold buffer A and the homogenate was consecutively filtered through nylon net filters with 60, 30 and 10 μm pore sizes (Merck Millipore, Tullagreen, Ireland). Following centrifugation (4,000 × g, 5 min, 4 C), the pellet of endosymbiotic bacteria was re-suspended in 400 μl of NaCl 0.85% supplemented with 20% glycerol as a cryoprotective agent, and stored at −80°C until flow cytometry analysis. Immediately prior to flow cytometry analyses, samples were thawed for 3 min at 42°C, vortexed and diluted to 1:20 with a NaCl 0.85% solution to reach the optimal cell concentration for flow cytometry analysis (<2,000 events/sec). All solutions were filter sterilized at 0.22 μm prior to use to avoid contamination with exogenous bacteria. For bacterial staining, 0.5 µl of the nucleic acid probe SYTO9 (3.34 mm; Molecular Probes Inc, Eugene, OR, United States) was added to 300 μl of the diluted bacterial suspensions. The mixture was then vortexed and incubated in the dark for 15 min at room temperature, following the manufacturer’s instructions. The stained samples were analyzed using a BD Accuri™ C6 flow cytometer (BD Biosciences, Franklin Lakes, NJ, United States) equipped with a blue laser (488 nm, air-cooled, 20 mW solid state) and cell green fluorescence was acquired with a photomultiplier tube detector and a 530 nm band-pass filter (503–563 nm). Flow cytometry measurements were run at low flow rate (14 μl/min) and the core stream was allowed to stabilize for 30 s prior to the 2 min acquisition. Cellular data were collected and processed using BD Accuri C6 software (version 1.0.264.21, BD Biosciences). The analyses were performed using logarithmic gains and specific detector settings, adjusted on non-stained samples to eliminate endosymbiont autofluorescence. For each developmental stage and condition, six biological replicates were processed.

### Aphid section preparation and histology

The antennae and legs were removed from appropriately staged aphids prior to fixation (eight aphids per condition and per stage). Aphids were fixed by immersion in PBS with 4% paraformaldehyde and 0.1% Triton X-100 at 4°C for 24 h, then transferred in PBS with 4% paraformaldehyde. After several washes in PBS, samples were embedded in 1.3% agar to facilitate manipulation then dehydrated through ethanol series (from 70% to absolute ethanol) before being moved to 1-butanol for 24 h at 4°C. Afterwards, aphids were embedded in Paraplast (McCormick Scientific LLC, Saint Louis, MO, United States) and sectioned at 3 μm thickness using a HM340E rotary microtome (ThermoScientific, Waltham, MA, United States). Sections were placed on polylysine-coated slides, dewaxed in methylcyclohexane and rehydrated through an ethanol series to PBS. Hematoxylin and Eosin (H&E) staining was performed using RAL products (RAL Diagnostics, Martillac, France) as previously described (Sapountzis et al., 2014), and sections were mounted with Diamount mounting medium (Diapath, Martinengo, Italia). Observations were made under transmitted light, on an Olympus IX81 microscope (Olympus Corporation) with 10X or ×60 lens magnification. Pictures were taken using an Olympus cooled color camera XC50 (Olympus Corporation) and CellSens software (Olympus Corporation).

### Transmission electronic microscopy

Bacteriocytes were isolated from the abdomen of appropriately staged aphids (60 aphids per condition and per stage) and directly fixed with 3% glutaraldehyde in sodium cacodylate buffer (0.1 M) for 24 h at 4°C. After washing thrice in sodium cacodylate buffer for 2 h, bacteriocytes were post-fixed in 1% osmium tetroxide for 60 min and contrasted in 1% uranyl acetate for 30 min in the dark. Samples were then dehydrated through a series of ascending ethanol solutions, from 30% to absolute ethanol, prior to incubation in a mix of absolute ethanol/propylene oxide (vol/vol) for 2 h. At each step, bacteriocytes were briefly centrifuged at low speed (300× g, 3 min, 4°C) and the supernatant was removed carefully with an elongated pipette tip before addition of the new solution. Bacteriocytes were embedded in Epoxy Embedding Medium (Merck KGaA, Darmstadt, Germany) with 1.7% benzyl dimethyl amine (BDMA) at 60°C for 72 h. From the resulting resin blocks, 70-nm-thick sections were cut using an UC7 Ultramicrotome (Leica) and were placed on 100-mesh copper grids (the minimum size allowing whole-bacteriocyte visualization). Ultrathin sections were contrasted with lead citrate for 7 min. Sections were observed with a 1,400 Flash transmission electron microscope (Jeol, Tokyo, Japan) at 80 keV.

### Statistics

Statistical analyses were carried out using the R software v4.0.5 (R Core Team, 2022) and *p*-values < 0.05 were considered significant. Survival data were analyzed with the survival and survminer R-packages and curves were compared using the Wilcoxon-Gehan rank test assuming a non-homogeneous survival probability during the course of the insect life cycle (Harrington and Fleming 1982). A global factorial linear model was used to analyze the effect of the different diets on the number of bacteriocytes, symbionts, and aphid weight at different stages of aphid development. Residuals’ heteroscedasticity was systematically tested. In case of homoscedasticity (artificial diets), a linear Gaussian model was used. When heteroscedasticity was observed (starvation), a generalized linearized model (GLM) was used with a quasi-Poisson distribution of residuals to consider their overdispersion (residual deviance/ddf >1). Post hoc multiple comparison probabilities were computed using Tukey’s correction when there were more than two comparisons involved in a same group of tests.

A mixed-effects model considering individual aphids as random factors was used to analyze the effect of diets on the surface of bacteriocytes at different developmental stages. For the starvation experiment, between-stages heteroscedasticity was introduced in the model whereas between-diets heteroscedasticity was not introduced as it did not significantly improve the likelihood. For the amino acid stress experiment, both between-stages and between-diets heteroscedasticities were introduced. Residuals’ heteroscedasticity was not observed, validating the use of a Gaussian model.

Cumulative fecundity curves were modeled using the three-parameter asymptotic model of Gompertz (Tjørve and Tjørve, 2017):

$$fecundity=K*\;e^{\log\left(\frac{N_{0}}{K}\right)e^{-r_{0}.\left(time-lag\right)}}$$

where K is the asymptote (i.e., the total number of laid nymphs), N0 is the number of nymphs at t = lag and r0 is the fecundity rate. Note that lag is not a parameter but corresponds to the beginning of the laying period. This non-linear function was used to fit individual aphid cumulative fecundity curves within a mixed model integrating the diet as a fixed factor (starvation, Phe and Leu experiments). As residuals did not show heteroscedasticity, generalized non-linear mixed models were not implemented. A summary of the models used in the studies, as well as ANOVA tables and post hoc tests are presented as Supplementary text in the Supplementary Material.

## Results

### Aphid life history traits under Leu or Phe stress

To assess the effect of depletion or excess of Leu or Phe on *A. pisum* performance, aphid survival and fecundity were recorded as metrics of their fitness.

**FIGURE 1:**
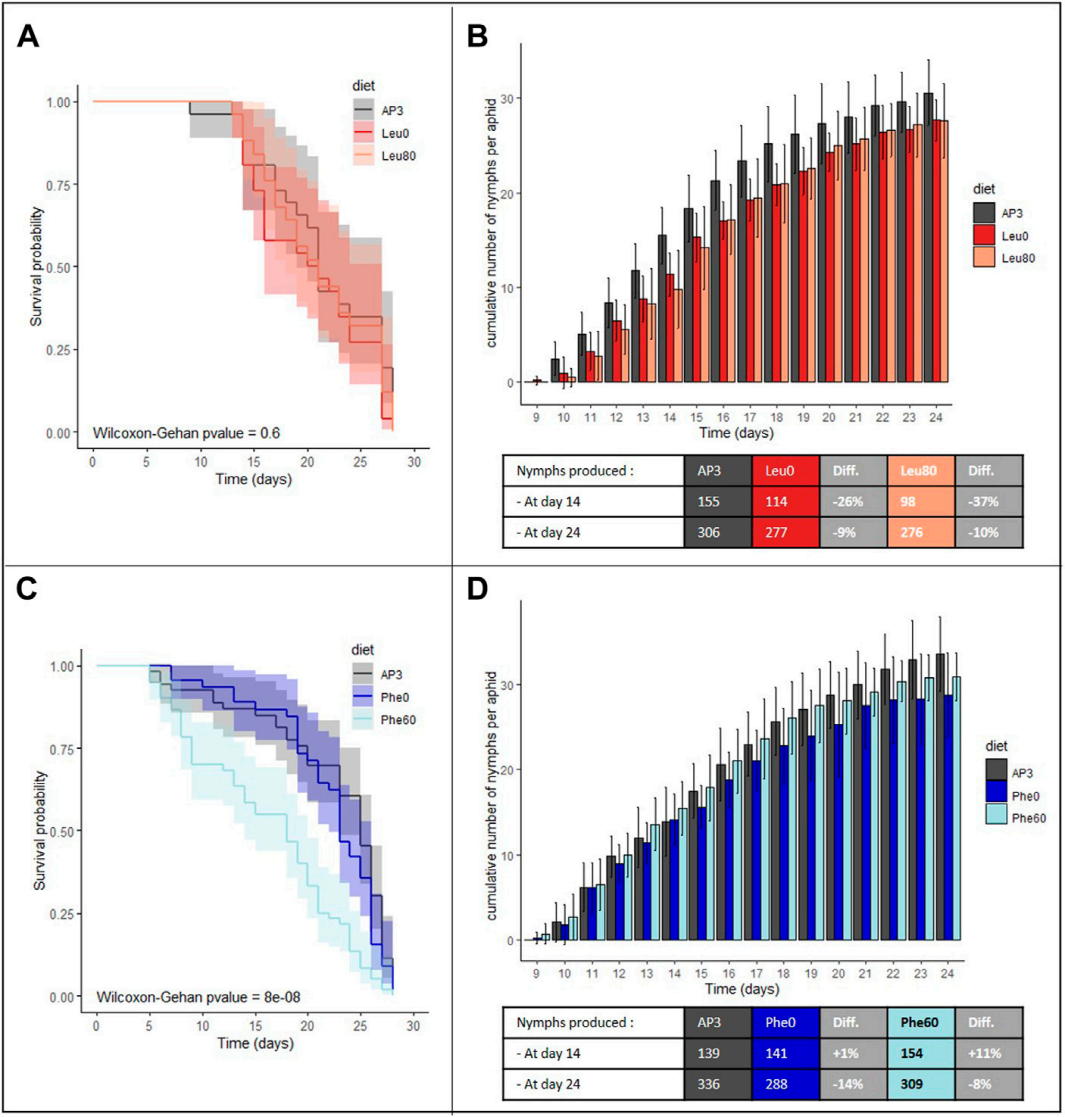
Impact of depletion or excess of leucine and phenylalanine on aphid survival and fecundity. **(A,C)** Survival analysis of *A. pisum* reared on the following artificial diets: a complete diet (AP3, dark gray), a leucine or phenylalanine depleted diet (Leu0, red or Phe0, dark blue) and a diet with leucine or phenylalanine excess (Leu80, orange or Phe60, light blue). For each diet group, survival curves are displayed with their predicted confidence intervals. Each diet group was composed of 60 aphids. Data were analyzed using the Wilcoxon-Gehan Test. **(B,D)** Cumulative numbers of progeny per aphid reared on the AP3, Leu0, Leu80, Phe0 or Phe60 diets, throughout the aphid reproductive phase. Results are reported as means (±SD) of 10 isolated individuals per diet. The number of cumulative nymphs produced at day 14 and day 24 for each diet are indicated beneath the corresponding graph with information on the difference between control and stressed population. Abbreviation: Diff, Difference.

Aphid survival probabilities were not significantly different when aphids were reared on a Leu depleted (Leu0) or enriched (Leu80) diet compared to the standard artificial diet AP3 (Figure 1A, *p* = 0.6). In those conditions, aphids could live up to 27 days and 50% mortality was reached after 21 days. However, Leu excess or deficiency both had a negative impact on aphid fecundity with a 9% and 10% reduction in offspring on the Leu0 and Leu80 diets, respectively, at the end of the reproductive period compared to the AP3 diet (Figure 1B; Supplementary Figure S2A **;** p (K_Leu0_-K_AP3_)<0.001 and p (K_Leu80_-K_AP3_) = 0.017). This reduction can be explained in part by the fact that aphids reared on these diets produced fewer nymphs daily than the AP3 controls, especially at the beginning of the reproductive period (p (N0_Leu0_-N0_AP3_)<0.001 and p (N0_Leu80_-N0_AP3_)<0.001). Indeed, at day 10, only four and three aphids on the Leu0 and Leu80 diets had started to lay nymphs compared to eight on the AP3 diet, and we counted 26% and 37% fewer nymphs on the Leu0 and Leu80 diet, respectively, at day 14. No significant difference in fecundity rate was observed between control and challenged aphids (Supplementary Figure S2A).

Contrary to what was observed for Leu, variation in Phe amount in the diet had a significant effect on aphid survival (Figure 1C, *p* = 10^–8^). While deficiency in Phe (Phe0 diet) had little to no impact, an excess of Phe (Phe60 diet) resulted in a significant increase in mortality (+23% on Day 8 compared to the AP3 control diet) and 50% mortality was reached after 16 days on the Phe60 diet against 24 days on the Phe0 and AP3 diets. This suggests that, in excess, Phe is toxic for aphids.

Aphids submitted to a depletion or excess of Phe produced 14 and 8% fewer nymphs than the controls at day 24, which was significant for Phe0 but not for Phe60 (Figure 1D; Supplementary Figure S2B; p (K_Phe0_-K_AP3_) = 0.026 and p (K_Phe60_-K_AP3_) = 0.286). Fecundity at the beginning of the laying period and fecundity rate were not significantly different between challenged and control aphids (Supplementary Figure S2B).

### Bacteriocyte dynamics under Leu and Phe stress

As regulation of bacteriocyte numbers has been shown to be a key response to depletion in Phe/Tyr in aphids (Colella et al*.*, 2018), we asked 1) whether dietary depletion or excess of a single amino acid (Leu or Phe) could induce changes in bacteriocyte dynamics throughout aphid development and 2) whether these variations depend on the applied stress.

**FIGURE 2:**
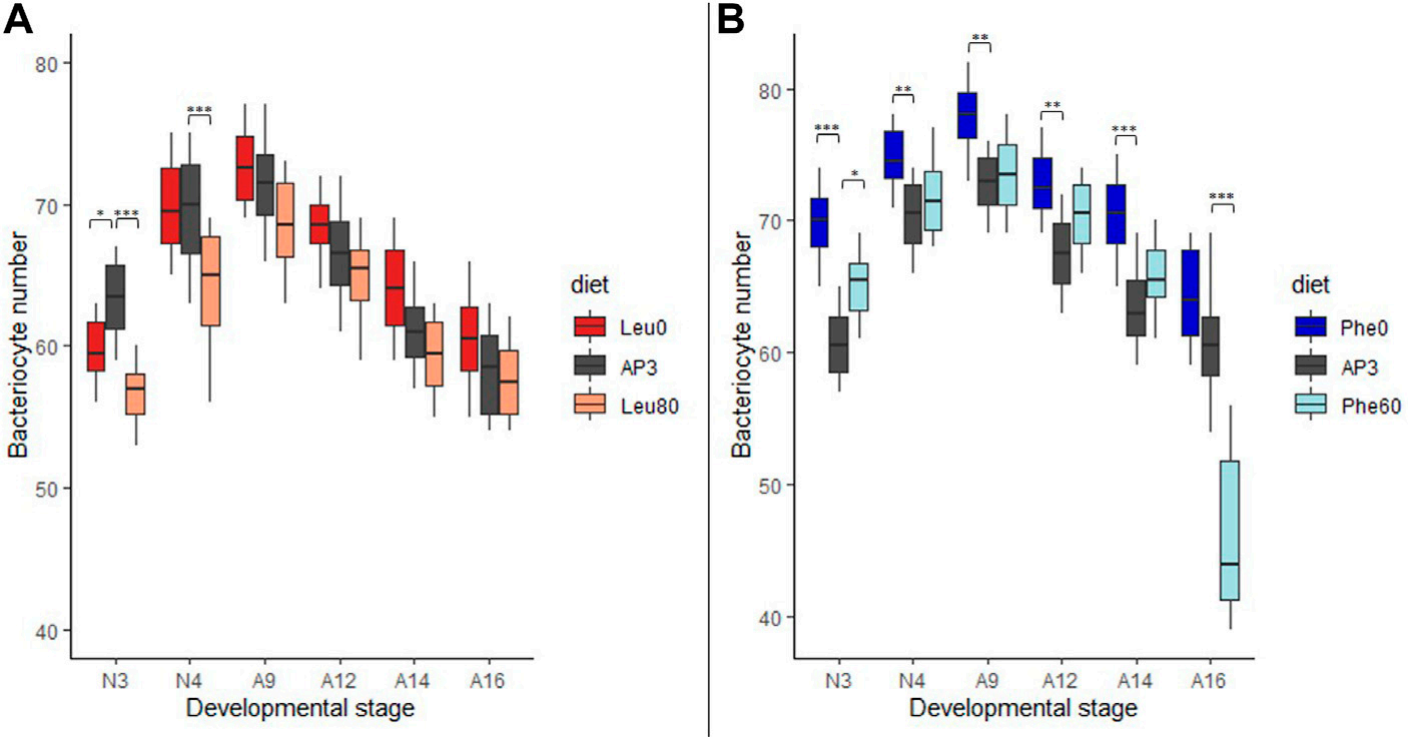
Impact of leucine and phenylalanine depletion or excess on bacteriocyte number. **(A)** Variation in the number of bacteriocytes per aphid reared on AP3 (dark gray), Leu0 (red) or Leu80 (orange) diets, in relation to host developmental stages. **(B)** Variation in the number of bacteriocytes per aphid reared on AP3 (dark gray), Phe0 (dark blue) or Phe60 (light blue) diets, in relation to host developmental stages. Results are displayed as box plots where central lines represent the medians, boxes comprise the 25–75 percentiles and whiskers denote the range; n = 10 aphids per stage and per condition, for a total number of 180 aphids dissected and analyzed. Data were analyzed with a factorial linear model followed by a post hoc multiple comparisons test (Tukey’s HSD test). Significant differences are indicated with asterisks (*, *p* < 0.05; **, *p* < 0.01). Abbreviations: N1 to N4, nymphal stages from 1 to 4; A9-A16, adult time points from day 9 to day 16.

Regardless of the diet on which the aphids were reared, the number of bacteriocytes systematically increased throughout nymphal development to reach a maximum at the beginning of adulthood, then progressively decreased as the insect aged (Figure 2; *p* < 0.001 when comparing the number of bacteriocytes between life stages for each diet). However, the magnitude of these variations, as well as the average number of bacteriocytes found in aphids at each developmental stage, was dependent on the stress they were submitted to.

Deficiencies in Leu or Phe had a significant effect on bacteriocyte numbers (p_AP3-Leu0_ = 0.012 and p_AP3-Phe0_<0.001). While the average number of bacteriocytes was significantly lower in N3 nymphs that were deprived of Leu (Leu0) than in control aphids (59.8 and 63.4, respectively; *p* = 0.03) this trend was later inverted as aphids at day 9 or older had more bacteriocytes on the Leu0 diet with, for instance, an average of 61.3 and 64.1 bacteriocytes in 14-day-old aphids reared on the AP3 and Leu0 diets, respectively (Figure 2A)**.** This results from the combination of two effects: 1) a greater increase in bacteriocyte number during nymphal development (+8.0 for AP3 and +12.90 for Leu0 between N3 and A9) and a smaller decrease during adulthood (-13.1 for AP3 and −12.2 for Leu0 between A9 and A16) for Leu0 compared to AP3. Phe deprivation also resulted in a higher number of bacteriocytes in Phe0 aphids compared to AP3. However, unlike what we observed with the Leu0 diet, this was true at all-time points (Figure 2B; *p* < 0.01 with the exception of A16) and the difference was largest in N3 nymphs (60.8 and 69.8 bacteriocytes, on average, for the AP3 and Phe0 diet respectively; *p* < 0.0001). Moreover, contrary to what we observed in case of Leu deprivation, deficiency in Phe led to a smaller increase in bacteriocyte number during nymphal development (+12.0 for AP3 and +8.1 for Phe0 between N3 and A9) and a greater decrease during adulthood (−12.0 for AP3 and −13.7 for Phe0 between A9 and A16). Globally, Phe deficiency had a stronger effect than Leu deficiency on bacteriocyte numbers with, on average, a difference of 5.7 vs. 2.0 bacteriocytes on the Phe0 diet and Leu0 diet respectively when compared to the AP3 diet and when considering all stages.

Excess in Leu or Phe also significantly impacted bacteriocyte numbers (p_AP3-Leu80_ < 0.001and p_AP3-Phe60_ = 0.004). The average number of bacteriocytes was lower in individuals fed on the Leu80 diet than in those fed on the AP3 diet at all time points (Figure 2A). These differences were more important for N3 aphids (63.4 and 56.7 bacteriocytes on average for AP3 and Leu80, respectively; *p* < 0.0001). Again, we observed a greater increase in bacteriocyte number during nymphal development (+8.0 for AP3 and +11.80 for Leu80 between N3 and A9), which was, however, not sufficient to make up the difference with AP3 aphids, and a smaller decrease during adulthood (−13.1 for AP3 and −10.9 for Leu80 between A9 and A16) for Leu80 compared to AP3. Contrary to what we observed in the case of Leu, excess in Phe did not lead to a decrease in bacteriocyte number. Instead, for each developmental stage, the number of bacteriocytes in Phe60 individuals was slightly higher than in AP3 ones, significantly so in N3 aphids (Figure 2B; *p* = 0.0032). The only exception was A16, with the average number of bacteriocytes in Phe60 aphids being significantly lower than in AP3 (*p* < 0.0001). Nevertheless, this result should be interpreted with caution, because half of the aphids had already died at this time point and the surviving aphids showed a darker cuticle indicative of oxidation and impending death.

Variation in Leu or Phe supply in the diet had little or no impact on bacteriocyte size. We did, however, observe a trend toward increased bacteriocyte size in aphids reared on the Phe0 diet compared to the AP3 control diet (Supplementary Figure S3). This was only significant at A9 and A12, where we also noted high variability between aphids reared on this diet.

A careful stereomicroscopical observation of internal organization of aphids submitted to Phe or Leu stresses showed no obvious morphological change in other tissues.

### Effects of starvation or total amino acid deprivation on aphid survival

**FIGURE 3:**
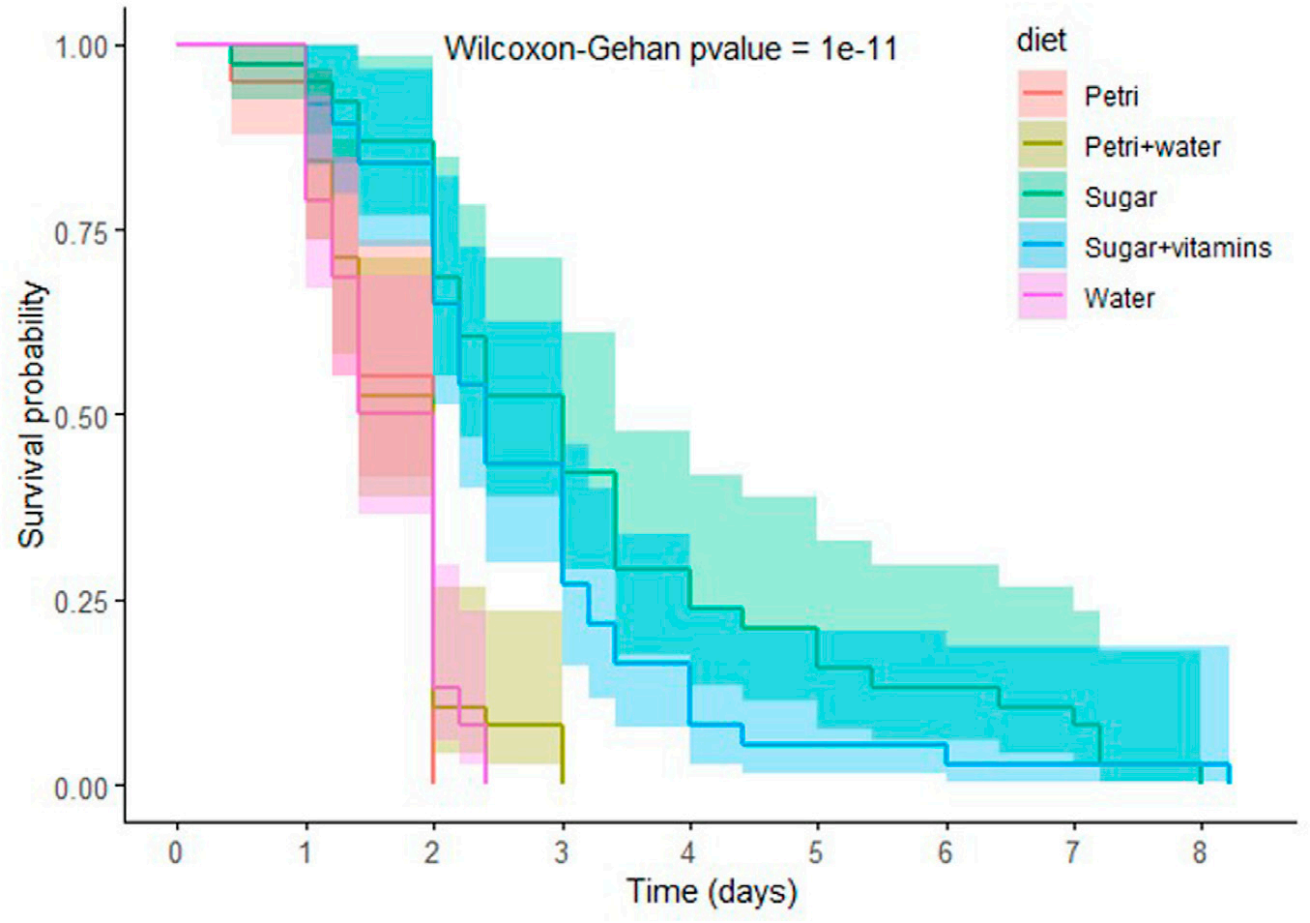
Impact of starvation and total amino-acid deprivation on aphid survival. Survival analysis of *A. pisum* reared in Petri dishes, without (Petri, red) or with a moist filter (Petri + water, sand), or in feeding chambers containing either water (Water, pink), sugary water (Sugar, green) or sugary water supplemented with vitamins (Sugar + vitamins, blue). Each diet group was composed of 40 aphids. Data were analyzed using the Wilcoxon-Gehan Test.

To further dissect the impact of amino acid depletion on aphid fitness and bacteriocyte dynamics, we decided to study more extreme conditions of nutritional stress hypothesized to induce more pronounced phenotypic changes in the challenged aphids. As a preliminary experiment, and in order to select the most promising stress conditions, we measured the survival rate of aphids that were placed in conditions of either complete starvation (consisting of Petri dishes with or without a moist filter paper, or feeding chambers containing only water) or total amino acid deprivation (consisting of feeding chambers containing 20% sucrose with or without the vitamins normally included in the AP3 diet) (Figure 3). The effect on survival was dependent on the stress applied and was significantly more potent when aphids were exposed to complete starvation (*p* = 10^–11^). Aphids reared on dry Petri dishes died in less than 48 h with 50% mortality reached after just 34 h. The addition of a moist filter inside the Petri dishes did not significantly improve aphid survival (*p* = 0.7), indicating that the mortality observed was not a consequence of desiccation. Aphids reared in feeding chambers and provided with water also died within 3 days. On the other hand, aphids that were fed sugar only reached 50% mortality after 3 days and could live for up to 8 days. Addition of vitamins to this diet did not significantly increase aphid survival (*p* = 0.2).

### Aphid life history traits following starvation for 1 day

Based on the results obtained when exposing aphids to various starvation conditions, we decided to study in more detail the impact of a one-day-long total-starvation period on aphid fitness and life history traits. We chose this condition because it caused the most drastic phenotypic change and it represents a stress that can readily happen in nature.

**FIGURE 4:**
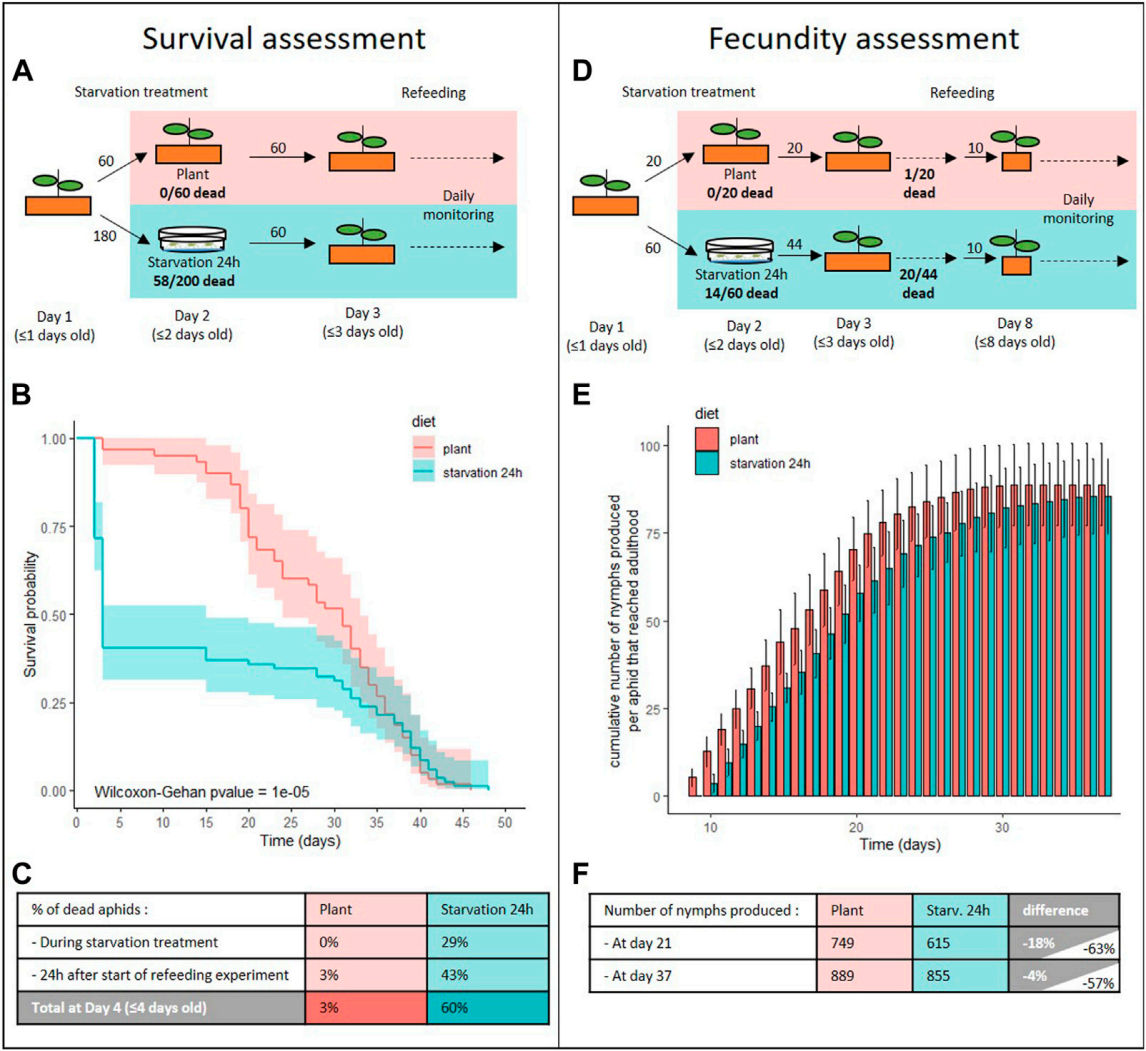
Impact of starvation stress on aphid survival and fecundity. **(A–C)** Survival analysis of *A. pisum* reared on plant from birth (plant, red) or that were starved for 24 h prior to being transferred to plant (starvation 24 h, cyan). **(A)** Experimental design and number of aphids used for the survival assessment. **(B)** Survival probabilities of aphids reared in each condition, with their predicted confidence intervals. Data were analyzed using the Wilcoxon-Gehan Test. **(C)** Information on the number of aphids that died during the starvation treatment and in the 24 h following transfer to plants. **(D–F)** Fecundity analysis of *A. pisum* reared on plant from birth (plant, red) or that were starved for 24 h prior to being transferred to plant (starvation 24 h, cyan). **(D)** Experimental design and number of aphids used for the fecundity assessment. **(E)** Cumulative numbers of progeny per control or starved aphid that reached adulthood, throughout the aphid reproductive phase. Results are reported as means (±SD) of 10 isolated individuals per diet. **(F)** Number of cumulative nymphs produced in the middle and at the end of the reproductive phase for starved and control aphids. Difference values on gray and white background were calculated considering only insects that reached adulthood or all individuals, respectively.

Twenty-nine percent of the aphids submitted to the 24 h starvation at N1 nymphal stage died before the end of the treatment (Figures 4A–C). Several of the surviving aphids presented clear signs of stress, such as muscular spasms and uncoordinated leg movements rendering them unable to actively move on the Petri dish. Following transfer to plants, starved aphids were also less mobile and took more time to get under the leaves compared to the control individuals. None of those signs were visible 48 h after this transfer. The impact of the 24 h starvation period was evident in aphids that survived the treatment as their survival after transfer to the plants was severely affected compared to the controls (Figures 4B,C **;** *p* = 0.05). Indeed, 43% of those aphids died within 24 h after transfer to plants, whereas no death was recorded in the control population during this period. Overall, 60% of the starved aphids died before day 4. In the following days, more deaths were recorded in the control population. Eventually, similar numbers of aphids remained in both populations at day 33 (21/60 and 20/60 aphids in control and stressed population, respectively) (Figure 4B).

The starvation treatment also had a significant impact on aphid fecundity (Figures 4D–F). First, 55% of the starved aphids included in the fecundity study never reached adulthood and therefore did not produce nymphs (Figure 4D). This alone explains a reduction in fecundity of starved aphids by more than half. Moreover, aphids that survived the starvation and reached adulthood began laying nymphs later than control aphids (Figure 4E; Supplementary Figure S4; p (N0 _starvation24h_-N0_plant_) = 0.0019). Indeed, all the control aphids that reached adulthood started to produce nymphs at Day 9 while starved aphids only started at Day 10. As a consequence, the cumulative number of nymphs produced per aphid was significantly lower in the starved population until day 21. However, starved aphids had an extended laying period (end of the laying period at 33 days and 29 days after birth for starved and control aphids, respectively; *p* = 0.016) that allowed them to reduce, but not completely make up, the difference with the controls after day 21. Overall, we observed an 18% reduction in the number of offspring produced by starved aphids at day 21 that was brought down to 4% by the end of the reproductive period (Figure 4F; Supplementary Figure S4; p (K _starvation24h_-K _plant_) = 0.7157).

We wanted to determine whether this reduced nymph number was due to a reduction in the number of embryos produced by the starved parthenogenetic mothers. Consistent with this hypothesis, stereomicroscopic observation of dissected young adults revealed poorly developed embryonic chains, which contained fewer and less well-developed embryos in starved aphids than in controls of the same age (Supplementary Figure S5). The embryonic chains of the stressed aphids were also noticeably more fragile and were consistently disrupted during dissection. In contrast, no obvious morphological change was observed in other tissues, such as the gut (Supplementary Figure S6). We also observed a delay in ecdysis timing (96% and 25% aphids of the control and stressed populations were adults at day 8, respectively; 100% and 82% at day 9;Supplementary Figure S7) as well as a significant weight reduction for the starved aphids compared to the controls (Supplementary Figure S8A). This weight reduction was more important in nymphs and young adults with an average reduction of 36% in N4 nymphs (*p* < 0.0001) and 50% in A9 adults (*p* < 0.0001). This effect was lessened to 7% in A23 aphids (not significant).

### Bacteriocyte and symbiont dynamics following starvation for 1 day

**FIGURE 5:**
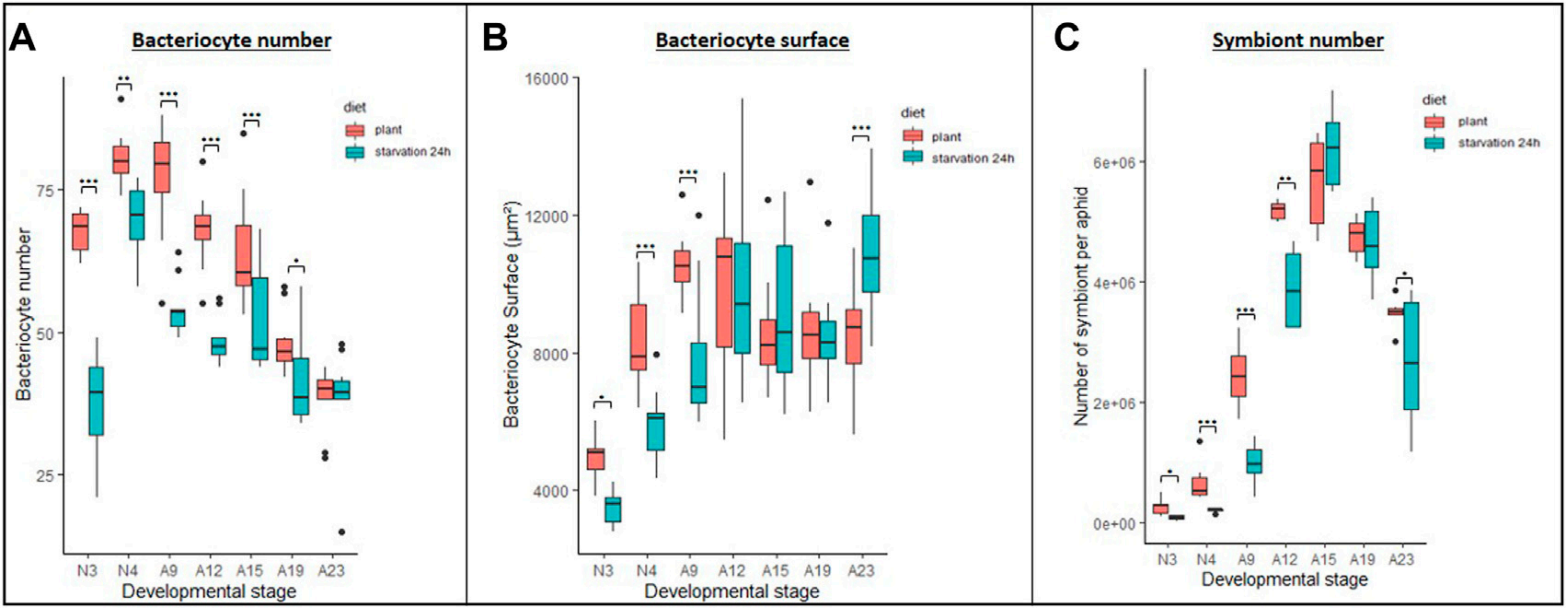
Impact of starvation stress on bacteriocyte and symbiont dynamics. **(A,B)** Variation in the number **(A)** and average surface **(B)** of bacteriocytes per aphid reared on plant from their birth (plant, red) or that were transferred back to plant after a 24 h starvation period (starvation 24 h, cyan), in relation to host developmental stage. Results are displayed as box plots where central lines represent the medians, boxes comprise the 25–75 percentiles and whiskers denote the range; *n* = 10 aphids per stage and per condition, for a total number of 140 aphids dissected and analyzed. Data on bacteriocyte numbers were analyzed with a generalized linear model followed by post hoc multiple comparisons tests. Data on bacteriocyte surfaces were analyzed with a mixed-effects model considering individual aphids as random factors followed by post hoc multiple comparisons tests. Significant differences are indicated with asterisks (*, *p* < 0.05; ***, *p* < 0.001). **(C)** Variation in the numbers of symbionts in *A. pisum* from the control (plant, red) or starved (starvation 24 h, cyan) population, in relation to host developmental stage. Symbionts were quantified by flow cytometry analysis of whole insects. Results are displayed as box plots where central lines represent the medians, boxes comprise the 25–75 percentiles and whiskers denote the range; *n* = 6 independent biological replicates per stage and condition (each biological replicate was composed of 10 aphids). Data were analyzed with a generalized linear model followed by post hoc multiple comparisons tests. Significant differences are indicated with asterisks (*, *p* < 0.05; **, *p* < 0.01). Abbreviations: N1 to N4, nymphal stages from 1 to 4; A9-A23, adult time points from day 9 to day 23.

To assess whether 24-h starvation early in life could have long lasting effects on bacteriocyte dynamics once the stress is removed, we recorded the number of bacteriocytes in starved and control aphids throughout their life cycle. We found that starved aphids younger than 23 days systematically had a lower number of bacteriocytes than the control aphids (Figure 5A; *p* < 0.05). For instance, in N3 nymphs, starved aphids presented on average 1.79-fold fewer bacteriocytes than the controls (*p* < 0.0001). However, this difference tended to diminish with time (1.15-fold in N4, *p* = 0.0049; 1.41-fold in A9, *p* < 0.0001; 1.39-fold in A12, *p* < 0.0001; 1.23-fold in A15, *p* = 0.0004; 1.16-fold in A19, *p* = 0.023) and we recorded a similar number of bacteriocytes in 23-day-old aphids. Only a weak correlation was found between bacteriocyte number and aphid weight (correlation coefficient of −0.3 and −0.17 for control and starved aphids, respectively). Aphids that had been starved for 24 h also showed a greater increase in bacteriocyte number during nymphal development, with a 1.78-fold increase between the N3 and N4 stages versus only 1.17-fold in controls. In later stages, bacteriocyte numbers decreased 1.32-fold between the N4 and A9 stages in starved aphids but remained the same in control aphids. Between stages A9 and A23, bacteriocyte numbers subsequently decreased by 1.35 and 1.99-fold, respectively, in starved and control aphids.

We also measured bacteriocyte surface (as a proxy for size) to determine whether there was a correlation between bacteriocyte number and size and whether this parameter was also impacted by nutritional stress. Initially, bacteriocytes were smaller in starved aphids, but become bigger in older individuals following a dynamics that is clearly distinct from the one observed for bacteriocyte numbers (Figure 5B; Supplementary Figure S9). More specifically, bacteriocyte surface was smaller in starved aphids at the N3, N4 and A9 stages (*p* = 0.038, 0.001 and 0.0003, respectively), similar between starved and control aphids at the A12, A15, and A19 stages (non-significant) and higher in starved aphids at the A23 stage (*p* = 0.001).

To assess whether there was a direct correlation between changes in bacteriocyte dynamics and the symbiont numbers within those cells, we quantified the population dynamics of symbionts using flow cytometry. Consistent with previous observations (Simonet et al., 2016a), we find that the *B. aphidicola* number varies significantly throughout the host life cycle, regardless of the condition (Figure 5C; *p* < 0.001). Both in starved and control aphids, the endosymbiont population drastically increases from N3 to A12 and starts to decrease thereafter. While the dynamics were similar, the total number of endosymbionts was significantly lower in starved nymphs and young adults compared to controls (*p* < 0.05).

### Phenotypic analysis of bacteriocytes in starved aphids

**FIGURE 6:**
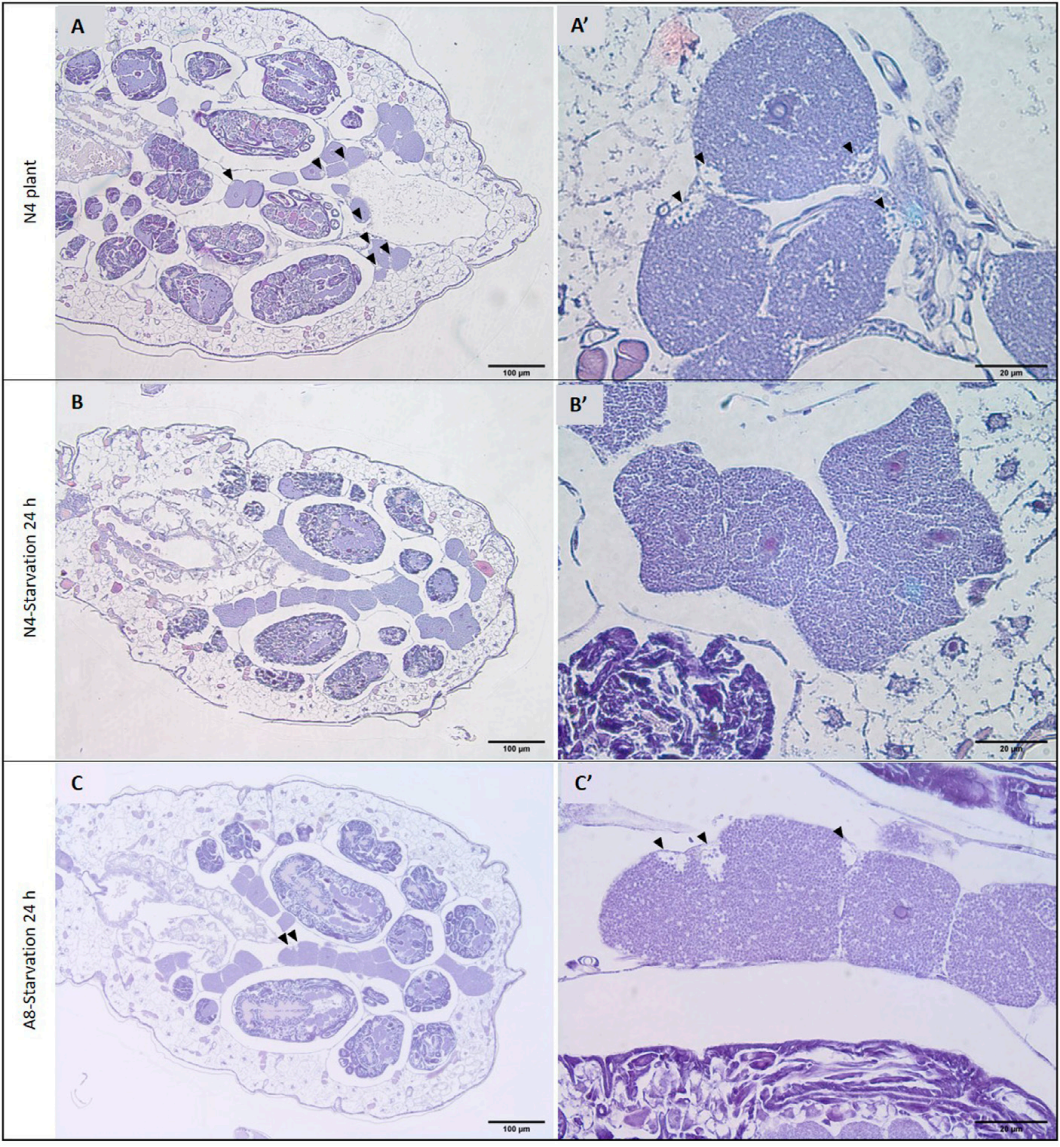
Effect of starvation on bacteriocyte phenotype. H&E staining of seven-day-old (N4 stage) aphids reared on plant from birth **(A)** or that were transferred back to plant after a 24 h starvation period **(B)**. H&E staining of eight-day-old (adult) aphids from the starved population **(C)**. **(A′–C′)** Higher magnification of representative bacteriocytes displaying low symbiont density zones. Arrowheads indicate low symbiont density zones that are characteristic of aphid bacteriocyte cell death.

Histological analyses of whole insect sections revealed a global developmental delay in starved aphids, with even young adults being smaller than N4 controls **(** Figures 6A–C)**.** This delay was also noticeable in the bacteriocytes. In N4 aphids, several bacteriocytes from the control individuals exhibited the low-symbiont density zones characteristic of aphids reared on plants previously reported in Simonet et al. (2016a). Low-symbiont density zones were not found in starved aphids of the same age (Figures 6A’,B’), but only appeared in 8-day-old individuals and in fewer bacteriocytes than controls (Figure 6C’).

**FIGURE 7:**
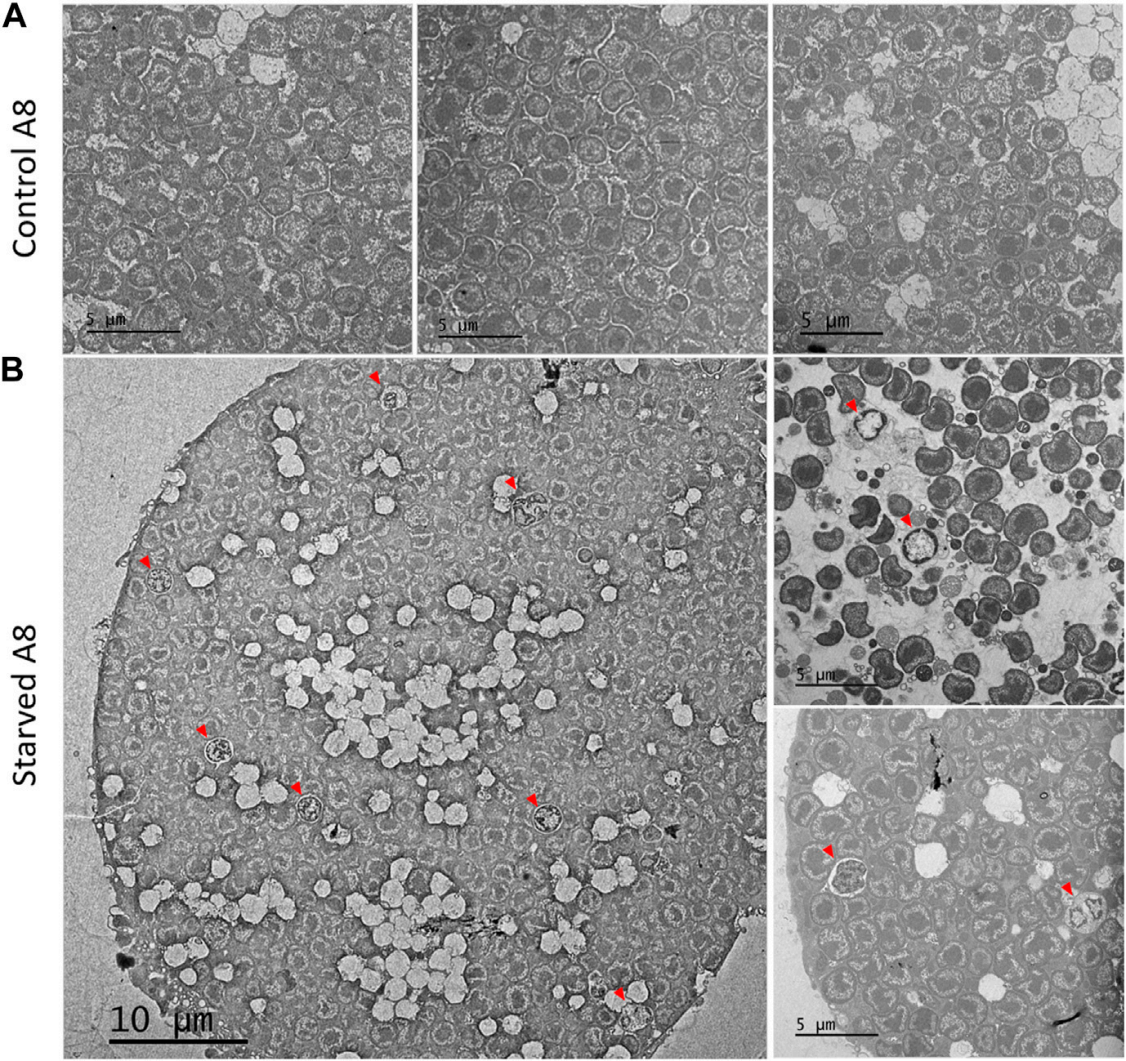
Effect of starvation on symbiont phenotype. TEM image of representative bacteriocytes from 8-day-old aphids reared on plant from birth **(A)** or that were transferred back to plant after a 24 h starvation period **(B)**. Red arrowheads indicate lysosomal-like vacuoles containing symbionts.

Electron microscopy analysis of isolated bacteriocytes revealed that a significant proportion of the bacteriocytes isolated from starved aphids (30% of the total number) contained symbionts seemingly undergoing lysosomal degradation (Figure 7). Compared to control aphids with bacteriocytes full of bacteria with normal appearance, the symbionts of the starved aphids often appeared malformed with DNA highly condensed in the whole cell, or confined to its periphery.

## Discussion

In the present work, we first demonstrated that aphids are able to quickly and efficiently adapt to excess or deficiency in essential amino acids. To do this, we used chemically defined artificial diets, systems that are less optimal for aphid growth than fresh plants (Liadouze et al., 1995), but provide the experimental versatility to study the impact of individual amino acids. We thus showed that depletion or excess in Leu or Phe have only a limited impact on pea aphid survival and fecundity, despite the fact that these two amino acids are considered essential for aphid physiology (Wilson et al., 2010). This points to the presence of robust cellular and molecular strategies to control metabolic homeostasis of these two amino acids in aphids.

For Phe, the resilience of aphids to its depletion was previously shown by Colella and others (Colella et al., 2018). Looking at a limited part of the aphid life cycle (from day 3–11), these authors demonstrated that one of the main responses of aphids to a co-depletion of Phe and Tyr is a transcriptional reprogramming of bacteriocytes, with increased expression of cell proliferation and growth-associated genes and increased bacteriocyte numbers. However, it was unknown whether this is a specific response to the lack of Phe and Tyr, or whether bacteriocytes would react in the same way in response to other depletions of essential amino acids, or to longer treatment periods. Here, we found that depletions in Phe or Leu, applied from the birth (day 0) to the peak in fecundity (day 16), systematically led to increased bacteriocyte numbers, suggesting that this is a global regulatory mechanism through which aphids respond to amino acid deprivation. We propose that the increase in bacteriocyte numbers allows the aphid to increase overall amino acid biosynthesis to compensate for the deficiency, potentially through an increased production of precursors by the endosymbionts. Indeed, both amino acids are produced by a tight collaboration between the host and the symbiont: *B. aphidicola* is able to synthesize all the precursors of Phe or Leu while the final step of the pathways depends on the host (Supplementary Figure S10) (Vellozo et al., 2011; Baa-Puyoulet et al., 2016). So, the interplay between the host and the symbiont is similar for these two biosynthetic pathways. Nevertheless, our results show that, while depletion of either Leu or Phe leads to increased bacteriocyte numbers, the response is much stronger (and statistically significant) for Phe.

This can be explained in several ways. First of all, several studies have shown that, in parthenogenetic aphids (like the ones we used in our study), embryos developing inside the aphid abdomen require a high amount of Phe and depend on the mother for the supply of this amino acid (Wilkinson and Ishikawa 1999; Bermingham and Wilkinson 2009; Bermingham and Wilkinson 2010). However, *B. aphidicola* does not seem able to regulate the genes involved in the production of Phe precursors neither during embryonic development (Bermingham et al., 2009) nor in response to Phe depletion (Reymond et al., 2006). On the other hand, the pea aphid genes involved in the conversion of the precursors to Phe are highly expressed or induced during parthenogenetic embryonic development (Rabatel et al., 2013), but not induced following Phe/Tyr depletion (Colella et al., 2018). Combined with our data, this suggests that bacteriocyte proliferation is a successful strategy, alternative to transcriptional regulation of the Phe biosynthetic pathway, to cope with Phe depletion and ensure that embryos develop properly. Contrary to Phe, Leu does not appear necessary for embryo growth (Bermingham and Wilkinson 2010) and the Leu biosynthetic gene LOC100167587 (encoded by the host genome) is not induced during embryonic development (Rabatel et al., 2013). However, Viñuelas et al. (2011) showed that this amino acid is essential for nymphal growth. Leu depletion (Leu0 diet) results in a 20% weight reduction and activation of the *de novo* biosynthesis of this amino acid at the end of the nymphal development. These authors also demonstrated that, unlike what was observed for Phe, *B. aphidicola* responds to Leu depletion by upregulating several genes involved in the biosynthesis of this amino acid: the chromosomal *ilvHI* operon and the plasmidic *leuABCD* operon. This response also encompasses an increase in copy number of the pLeu plasmid which harbors the *leuABCD* operon. This capability of *B. aphidicola* to respond quickly to Leu depletion could be a reason why we did not observe a large increase in the number of bacteriocytes following a Leu depletion. Plasmid regulation was previously reported to be maximal during the first 2 days after the beginning of the treatment but the levels of transcriptional and pLeu copy numbers induction returned to basal values after 7 days (Viñuelas et al., 2011). In the current study, the increase in bacteriocyte number in response to the Leu0 diet was observed starting from day 9 and until the end of the experiment. All these data suggest that different strategies are used by the pea aphid to cope with Leu depletion with a prominent molecular response of the symbiont at first, followed by a cellular response manifested by the proliferation of bacteriocytes. The existence of stress-specific regulatory mechanisms is further supported by the observation that excess in Leu resulted in decreased bacteriocyte numbers. This observation is consistent with what would be expected as, in this context, most of the host Leu requirements would already be covered by its dietary intake, thus reducing the need for Leu neosynthesis and bacteriocyte number increase. Moreover, Leu has been shown to display a stimulatory effect on aphid feeding (Viñuelas et al., 2011), which would result in higher levels of all nutrients in the aphid hemolymph and further reduce the need for bacteriocyte proliferation. In the case of Phe excess, we observed an initial increase in bacteriocyte number followed by a sharp drop at day 16. Interpretation of these results is complicated by the fact that the concentration we used here (Phe60 diet) is toxic for aphids and able to provoke a significant and early increase in mortality compared to the AP3 control diet. Toxicity of Phe has previously been documented in mammals where it has been demonstrated that this amino acid is neurotoxic, in particular in the context of a metabolic disease called phenylketonuria (van Spronsen et al., 2009; Adler-Abramovich et al., 2012; Schuck et al., 2015).

The results presented above show that aphids are resilient to amino acid stress with changes in bacteriocyte number being one strategy to compensate for the stress encountered. Since these stress responses are induced by concentration changes in single amino acids, we asked what would be the consequences of more global nutrient deprivation. After testing different treatments, we chose to submit neonate nymphs (<1 day old) to starvation for 24 h followed by refeeding. This stress can readily occur in nature when aphids fall from their host plant due to overpopulation or in response to predators. Furthermore, 24 h starvation had a profound impact on aphid physiology while still yielding enough surviving aphids for downstream analyses.

Twenty-four hours starvation resulted in high initial mortality, with 60% of aphids dying before they were 4 days old (N3 stage). This highlights the extreme susceptibility of neonate aphids to starvation, something previously documented in soybean aphids where survival was reduced by 66.5% following starvation for 48 h (Zhang et al., 2015). The response of aphids to starvation appears age-dependent as adult aphids are able to survive for longer periods of time, up to 8 days, when deprived of food (Stadler et al., 1994; Xu et al., 2012; Xu et al., 2019), possibly due to their lower physiological requirements compared to developing nymphs. The starvation period also caused an overall delay in the physiology of the surviving parthenogenetic mothers, which was seen at different levels. We observed a significant reduction in aphid growth and a 24 h delay in the transition to adulthood. These aphids also exhibited poorly developed embryonic chains compared to aphids of the same age that were fed throughout their entire life, as well as a delay in reproduction, which started later but lasted longer. Those data are consistent with previous reports of delayed reproduction (Brough and Dixon 1990; Kouamé and Mackauer 2012; Xu et al., 2012), and in some cases selective reabsorption of embryos (Ward and Dixon 1982; Stadler 1995), in aphids exposed to starvation. This suggests that aphids are able to reduce reproductive investment to increase their survival probability when confronted with insufficient food, as seen in other insects (Bell and Bohm 1975; Kotaki 2003; Terashima and Bownes 2004; Kajita et al., 2009; Billings et al., 2019). The physiological delay was also evident in the bacteriocytes, where low-symbiont density zones, a characteristic of the onset of aphid bacteriocyte cell death (Simonet et al., 2016a; Simonet et al., 2018), appeared later than in control aphids. Overall, development of the surviving aphids appears to have been paused during starvation, leading to a delay before functional readouts reach levels comparable to those of control aphids.

Based on the results observed in the absence of Phe or Leu (*i.e.* increase in bacteriocyte numbers) one could have expected that a stronger nutritional deprivation, such as a 24 h starvation period, would lead to an even stronger increase in bacteriocyte number. However, we found that this treatment generates a drastic decrease in bacteriocyte numbers, which is particularly striking in young nymphs, and a reduction in bacteriocyte size. This indicates that bacteriocyte proliferation is not an inherent response to nutrient deprivation. Importantly, several insects, in particular holometabolous insects such as *Drosophila melanogaster*, have been shown to slow or completely arrest the growth of specific tissues during periods of starvation (Tang et al., 2011). It is therefore possible that, under such drastic nutritionally adverse conditions, aphids reduce investment in bacteriocyte proliferation to favor other physiological processes that are more essential for their survival and growth. The reduction in the size and number of bacteriocytes was accompanied by a reduction in the total number of symbiotic bacteria. While this can be directly explained by the decrease in bacteriocyte number and size, we cannot exclude that active processes of symbiont degradation or inhibition of bacterial division take place. *B. aphidicola* needs energy to replicate its genome, divide, to ensure its own metabolism and participate in the symbiotic metabolism (Wilson et al., 2010). Under starvation, endosymbiont replication and division could therefore be stunted, which might contribute to the lower number of *Buchnera* found in aphids following starvation. Bacteriocytes, and more specifically their bacterial content, could also be actively recycled as an additional energy source for the starved aphids. Several earlier reports support this hypothesis. For instance, Hongoh and Ishikawa (1994) demonstrated that young adult aphids that had completed their final ecdysis 2 days before and were submitted to a 3-day starvation regimen exhibited a drastic decrease in total bacteriocyte volume (number and size), which, at least in the case of winged aphids, was followed by a rapid increase when starved individuals were transferred back to host plants. More recently, it was shown that when adult soybean aphids are starved for 36 h, the transcriptome of *B. aphidicola* shows up-regulation of genes involved in the metabolism of peptidoglycan (murein), a component of the bacterial cell wall, and a down-regulation of endosymbiont genes coding for the flagellar hook and basal body (Enders et al., 2014), which have been suggested to have a role in protein transport between bacterium and host (Maezawa et al., 2006). The authors propose that those results either reflect impaired functioning of *Buchnera* under stress and/or an overall reduction in bacterial titer levels that could involve a bacterial turnover in stressed populations. Interestingly, our electron microscopy analysis revealed that symbionts from starved aphids are being degraded within bacteriocytes of young adults, a process that likely involves the lysosomal system. Such lysosomal figures had previously only been documented in older aphids during the later stages of bacteriocyte cell death (Simonet et al., 2018), where it was hypothesized that it could be a mechanism to reduce the physiological cost of maintaining the symbionts in degenerating cells and also to recycle *B. aphidicola*. In light of all these data, we propose that when aphids are starved, the lysosomal system of bacteriocytes may be activated to actively digest endosymbionts as an alternative energy source.

Of note, regardless of the condition under which aphids were reared in this study (i.e. rearing on plants with or without starvation or on artificial media with or without amino acid stress) and despite the changes reported above, we observed a consistent pattern of variation in bacteriocyte numbers with developmental age. We observed an increase during nymphal development and a decrease after the onset of adulthood, a pattern correlated with the physiological requirements of aphids that are particularly high prior to the last molt. This suggests that, even under nutritional stress, a basal regulatory mechanism remains in place to ensure bacteriocyte homeostasis, to which is added the modulation of bacteriocyte numbers in response to stress. Importantly, after they were transferred back to plants, the differences in bacteriocyte number, size and bacterial load between starved and control aphids progressively diminished as the insects aged. This observation further supports the notion of cellular and molecular strategies that participate in the fine-tuning of bacteriocytes in response to the physiological needs of the aphid. Based on our results, these strategies include a modulation of bacteriocyte proliferation, which is activated in starved aphids so that bacteriocyte number rapidly increases during nymphal development, and a global slow-down of bacteriocyte cell death, with a more gradual decrease in adults.

This work represents a first step towards understanding how bacteriocyte dynamics are integrated in and contribute to the adaptation of a symbiotic insect to its environment and highlights the high physiological plasticity of these cells in response to the host nutritional status. Further studies are now needed to characterize the cellular and molecular mechanisms involved in this plasticity as well as the signals linking nutrient sensing to bacteriocyte dynamics. This could involve, in particular, Dual-RNA-seq analyses to identify aphid and *Buchnera* genes that are differentially expressed under trophic stress conditions and might be involved in the regulation of the bacteriocyte and symbiont dynamics. An *a priori* approach should also be used to investigate the possible role of selected peptides (*e.g.* Insulin Related Peptides) and hormones that are known to act in metabolic regulation in other insects, for instance by allowing them to regulate their growth and that of their organs in response to nutritional stress (Tang et al., 2011; Caers et al., 2012; Okamoto and Yamanaka 2015; Chowanski et al., 2021; Huygens et al., 2022).

## Data Availability

The original contributions presented in the study are included in the article/Supplementary Material, further inquiries can be directed to the corresponding authors.
