# Supplementary material for: Bacteriocyte plasticity in pea aphids facing amino acid stress or starvation during development
Source: Front Physiol. 2022 Nov 10;13:982920. doi: 10.3389/fphys.2022.982920 (PMC9685537; doi:10.3389/fphys.2022.982920)
Supplement: Supplementary file 1 [file DataSheet1.PDF]

Supplementary Material

Supplementary Figures

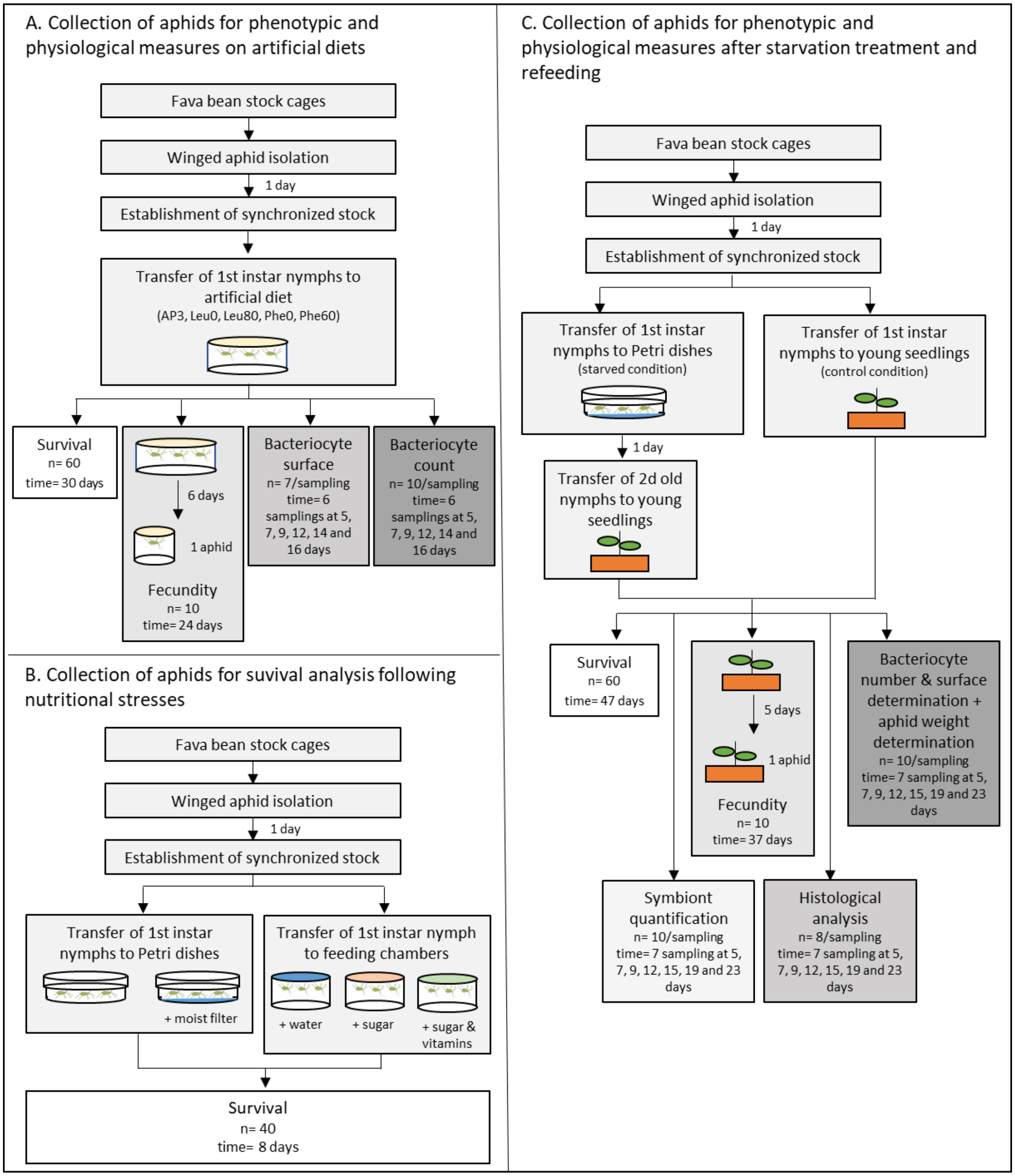

Supplementary Figure 1. Experimental design used for phenotypic and physiological measures on artificial diet (A), following starvation treatments (B) and after starvation and refeeding (C).

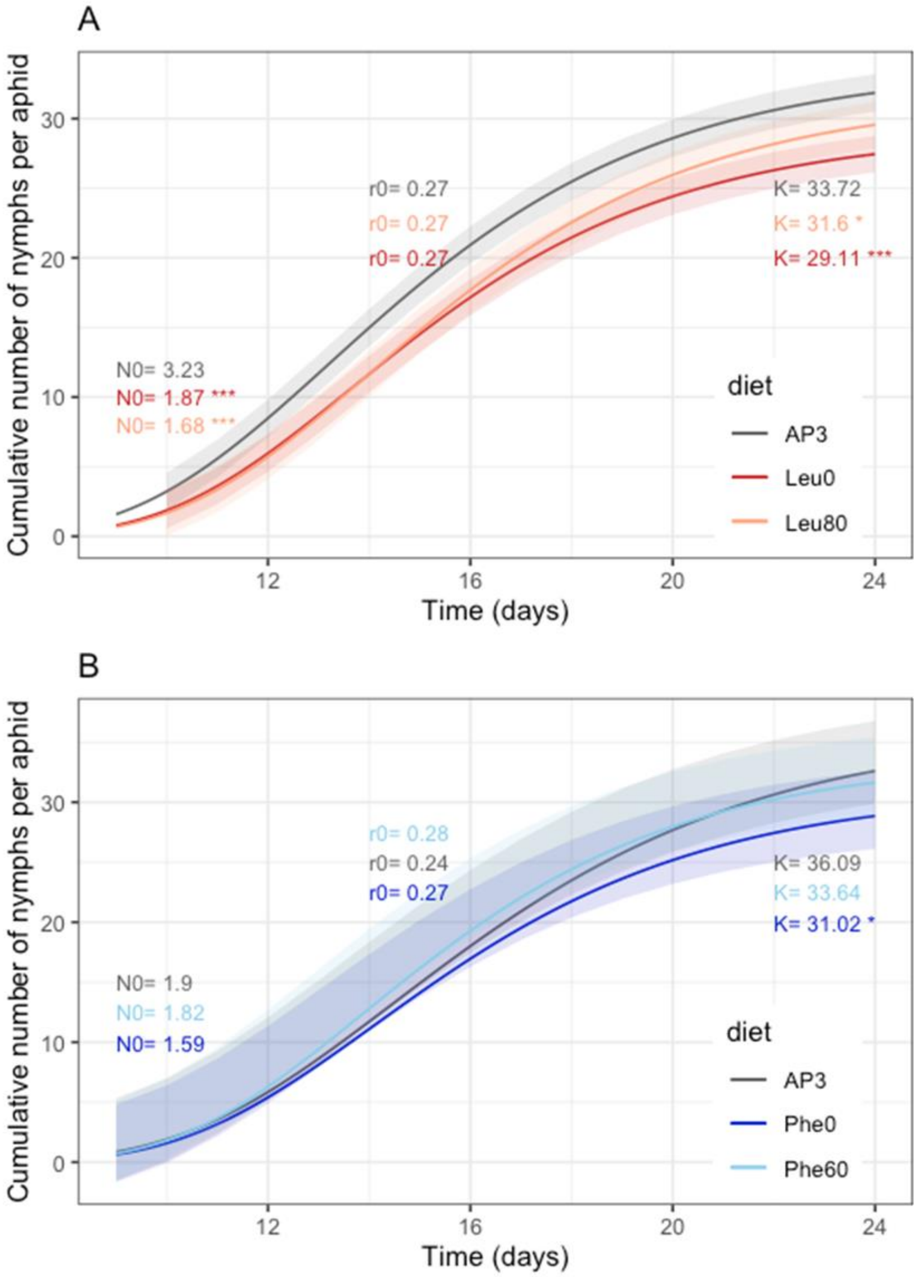

Supplementary Figure 2. Impact of leucine and phenylalanine depletion or excess on aphids' fecundity. (A) Modeling of the cumulative numbers of progeny per aphid reared on AP3 (dark gray), Leu0 (red) and Leu80 (orange) diets using a mixed model with individual aphids as random effects and diets as fixed effects. (B) Modeling of the cumulative numbers of progeny per aphid reared on AP3 (dark gray), Phe0 (dark blue) and Phe60 (light blue) diets using a mixed model with individual aphids as random effects and diets as fixed effects. Parameters of the fitted Gompertz curves are given following the color code of each graph with asterisks indicating the significance of their difference with the control AP3 (*pvalue<0.05, ***pvalue<0.001). Results from the corresponding tests are detailed in the Supplementary text. The colored zones represent the 95% confidence intervals around the curves.

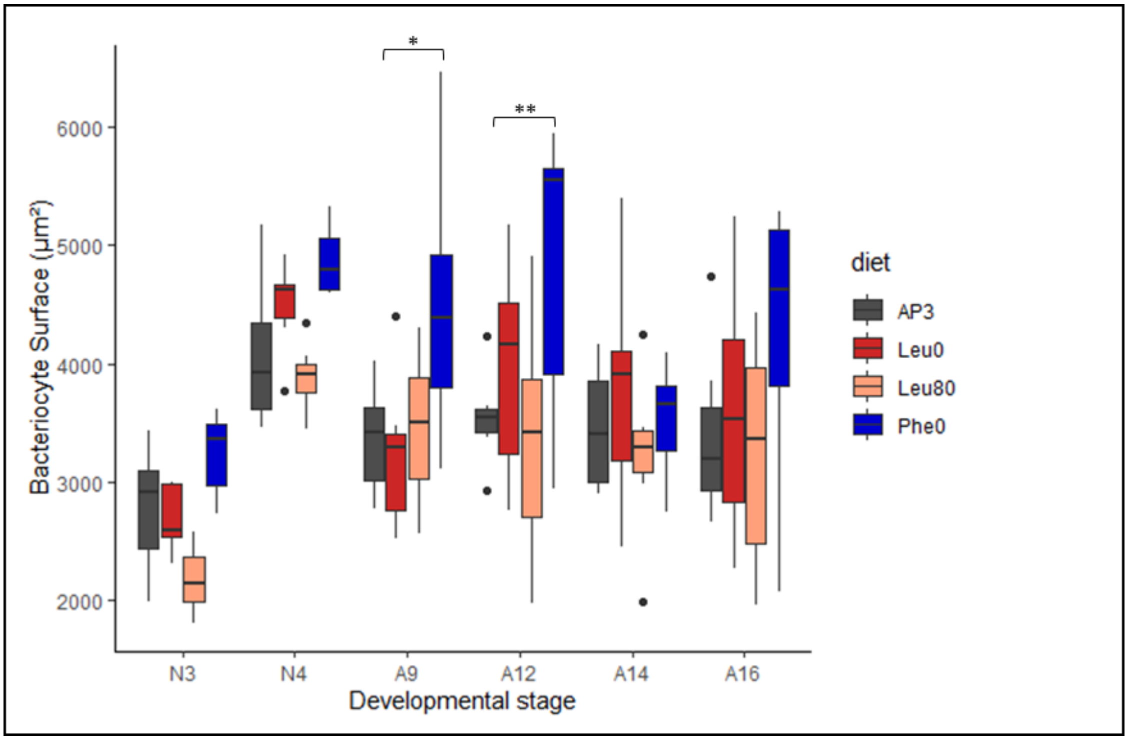

Supplementary Figure 3. Impact of leucine and phenylalanine depletion or excess on bacteriocyte surface. Variation in the average surface of bacteriocytes per aphid reared on the following artificial diets: a complete diet (AP3, dark gray), a leucine or phenylalanine depleted diet (Leu0, red or Phe0, dark blue) and a diet with leucine excess (Leu80, orange), in relation to host developmental stages. Results are displayed as box plots where central lines represent the medians, boxes comprise the 25–75 percentiles and whiskers denote the range; n = 7 aphids per stage and per condition, for a total number of 168 aphids dissected and analyzed. Data were analyzed with a mixed effect model considering individual aphid as random factor followed by a post hoc multiple comparisons test. Significant differences are indicated with asterisks (*, P< 0.05; **, P<0.01). Abbreviations: N3 and N4, nymphal stages 3 and 4; A9-A16, adult time points from day 9 to day 16.

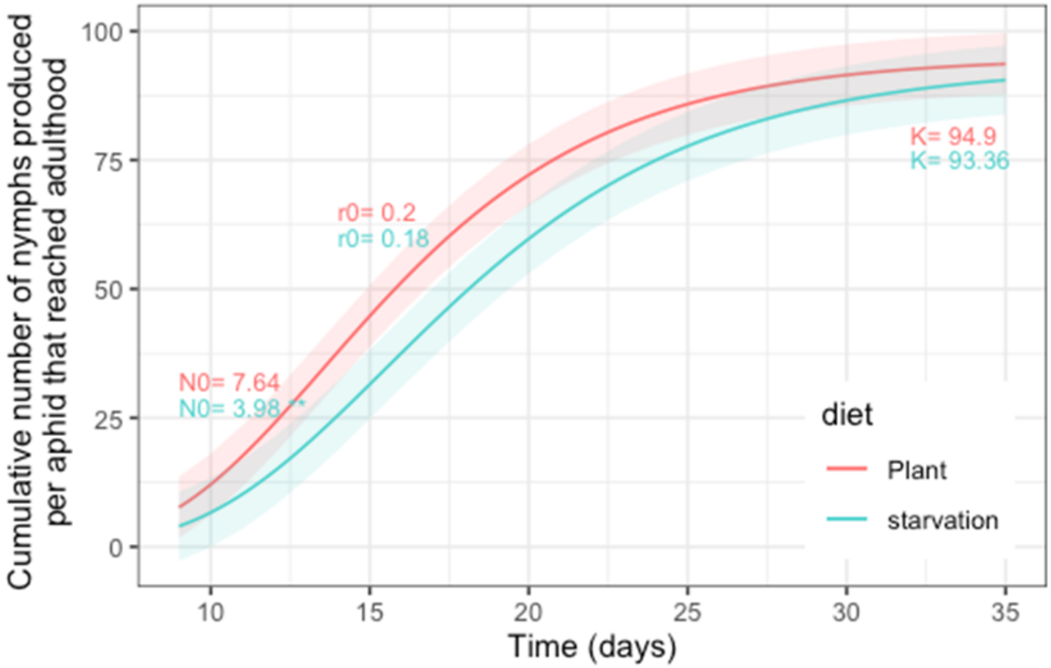

Supplementary Figure 4. Impact of starvation on aphids' fecundity. Modeling of the cumulative numbers of progeny per aphid reared on plant (red) or that were starved for 24 h prior to being transferred to plant (cyan) using a mixed model with individual aphids as random effects and diets as fixed effects. Parameters of the fitted Gompertz curves are given following the color code of each graph, with asterisks indicating the significance of their difference with the plant (**pvalue<0.01). Results from the corresponding tests are detailed in the Supplementary text. The colored zones represent the 95% confidence intervals around the curves.

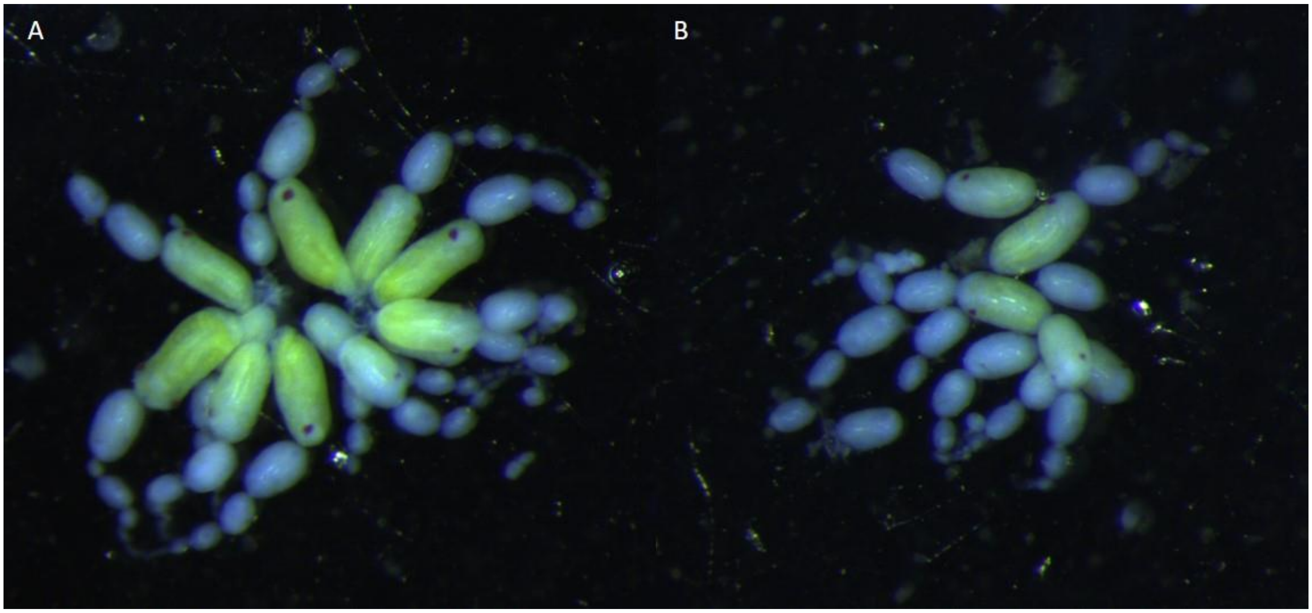

Supplementary Figure 5. Morphological changes of embryonic chains following starvation. Embryonic chains were dissected from seven-day-old aphids (N4 stage) from control (A) and starved (B) populations. Magnification x8.

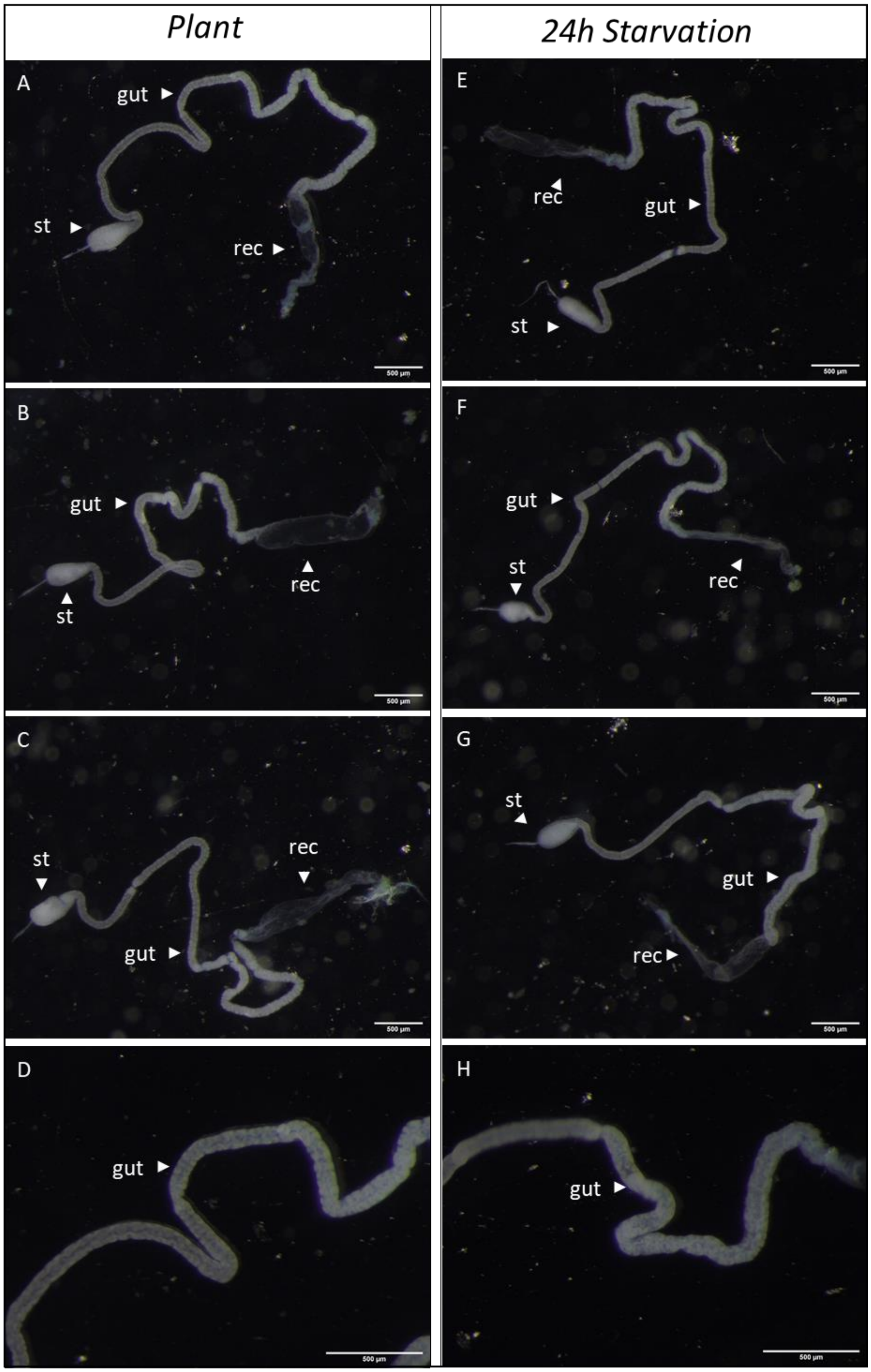

Supplementary Figure 6. Absence of gross morphological changes in aphid gut following starvation. Representative images of gut from seven-day-old aphids (N4 stage) from control (A-D) or starved (E-H) populations. Abbreviations: st, stomach; re, rectum.

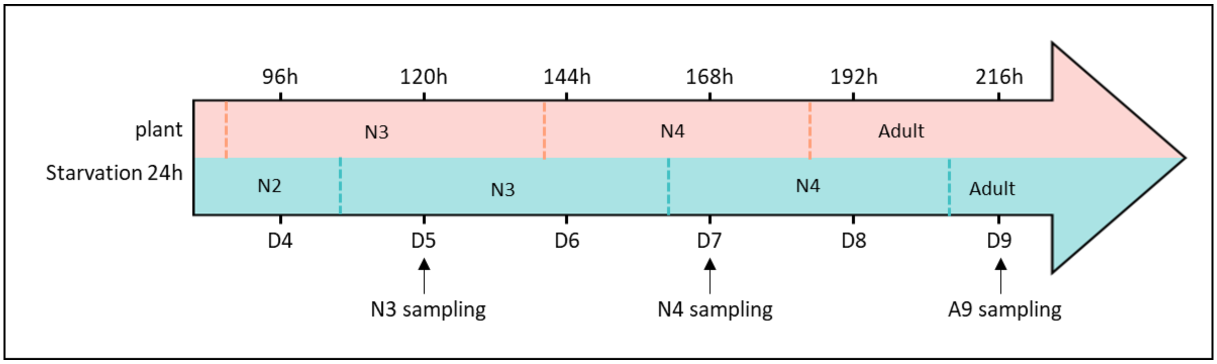

Supplementary Figure 7. Impact of starvation on aphid molting. Timeline of molting phases for pea aphids reared on plant from birth (plant, red) or that were starved for 24 h prior to being transferred to plant (starvation 24 h, cyan). The dotted lines indicate the average age at which the aphids underwent their second, third and fourth molts (from left to right) for each condition. Abbreviations: D4 to D9, Day 4 to 9, respectively.

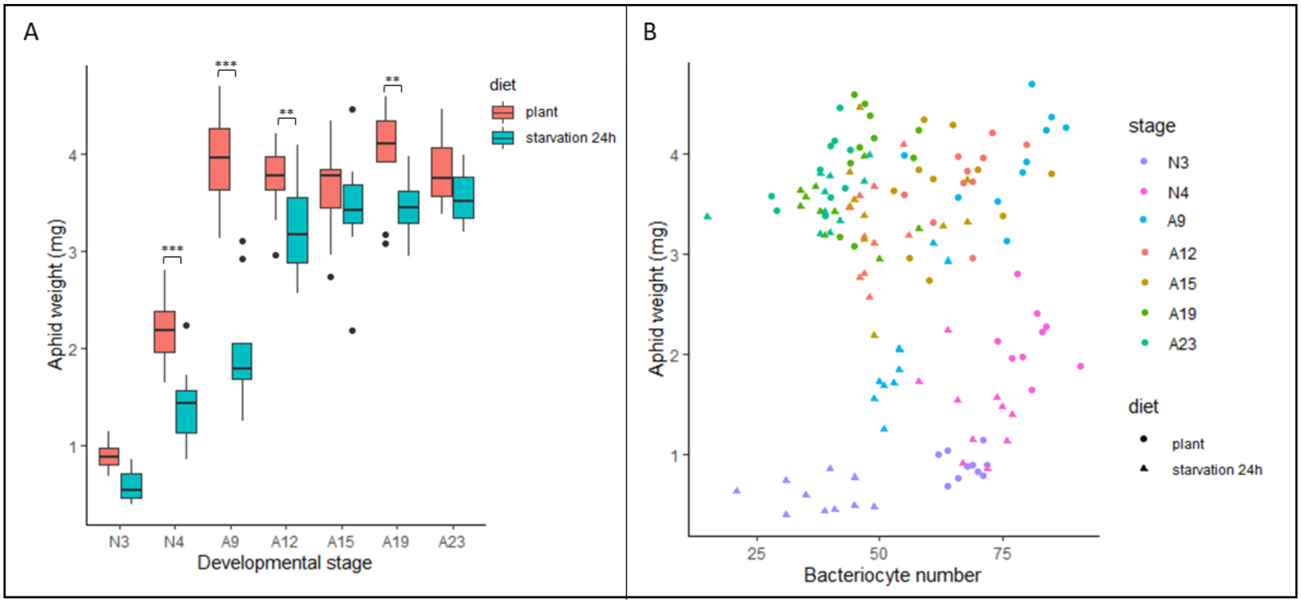

Supplementary Figure 8. Impact of starvation on aphid weight. (A) Aphid weight in relation to host developmental stage. Results are displayed as box plots where central lines represent the medians, boxes comprise the 25–75 percentiles and whiskers denote the range; n = 10 aphids per stage and per condition. Data were analyzed with a factorial linear model followed by post hoc multiple comparisons tests. Significant differences are indicated with asterisks (**, P<0.01, ***, P<0.001). (B) Relation between aphid weight and bacteriocyte number in aphids from the control (circle) and starved (triangle) population, in relation to host developmental stage. Abbreviations: N1 to N4, nymphal stages from 1 to 4; A9-A23, adult time points from day 9 to day 23.

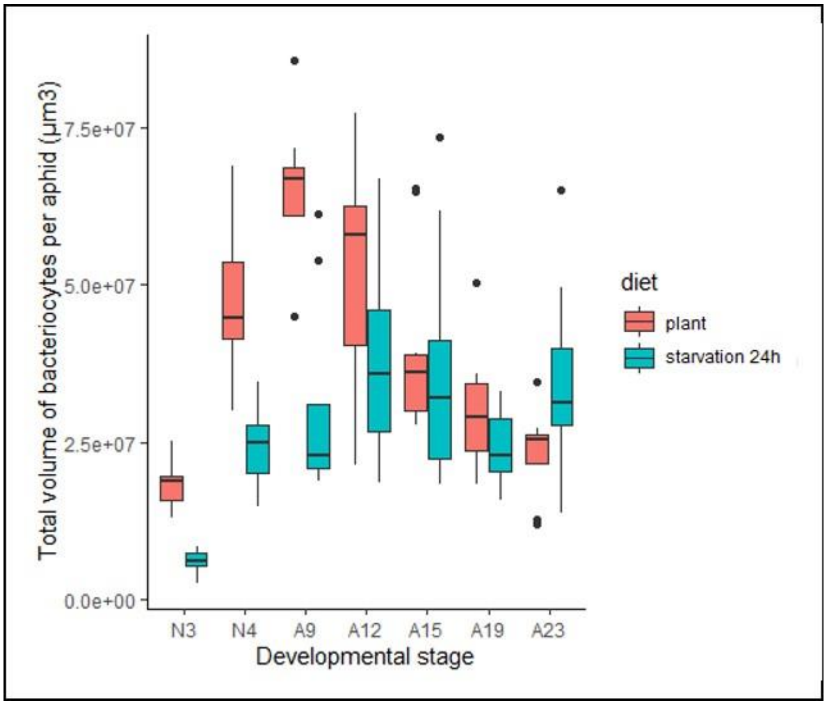

Supplementary Figure 9. Impact of starvation on total bacteriocyte volume. Total bacteriocyte volume was calculated by multiplying the average volume of bacteriocytes in each aphid by the number of cells counted at each stage. To obtain an estimate for cell volume, each bacteriocyte was treated as a sphere and the bacteriocyte volume value (V) was calculated applying the standard formula: $V = \frac{4}{3}\sqrt{\frac{A^3}{\pi}}$ with (A) the area of bacteriocytes. Abbreviations: N1 to N4, nymphal stages from 1 to 4; A9-A23, adult time points from day 9 to day 23.

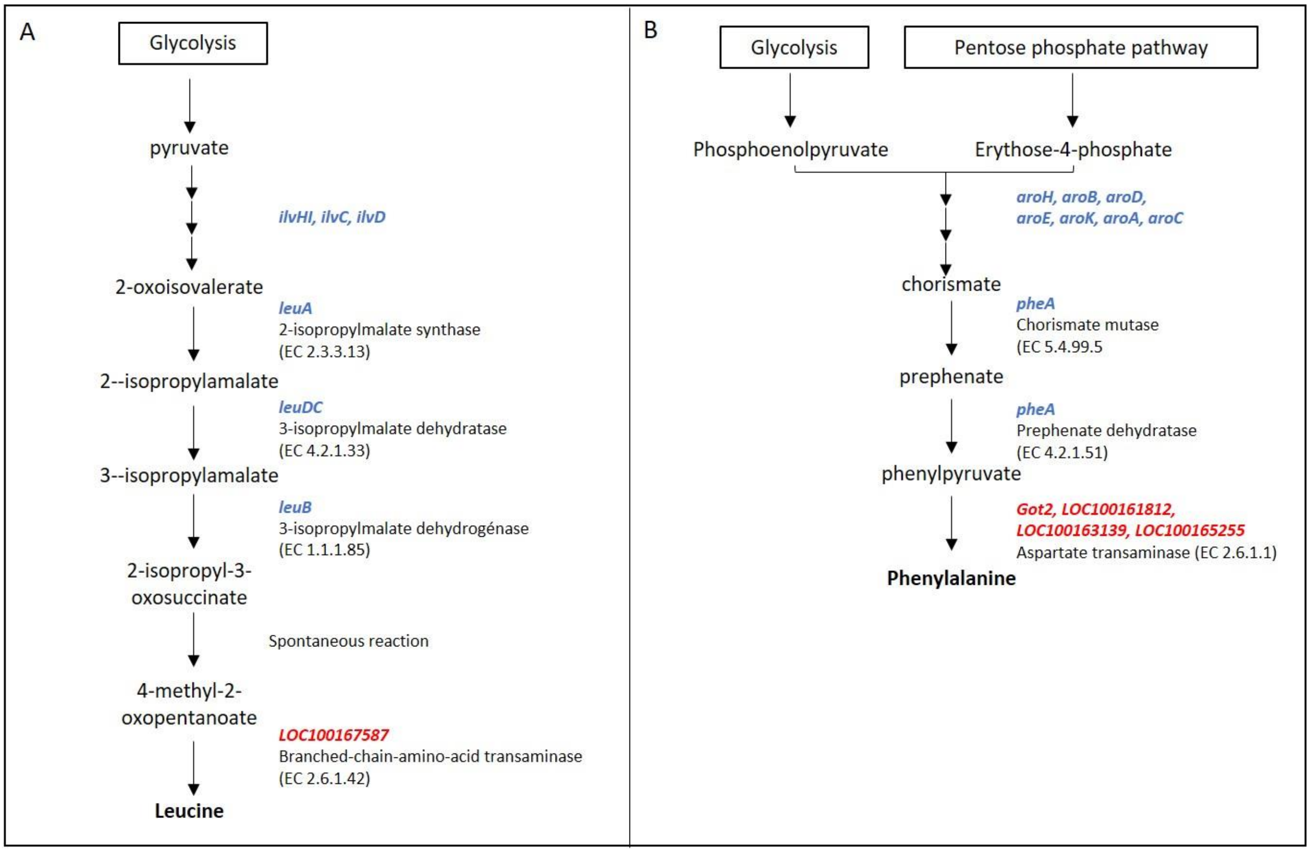

Supplementary Figure 10. Pathways for the biosynthesis of Leucine (A) and Phenylalanine (B). The pathways were modified from http://acypicyc.cycadsys.org/ and completed with data from Wilson and colleagues (2010). The enzymes encoded by *B. aphidicola* or the pea aphid are highlighted in blue and red, respectively.

Supplementary Table 1. Composition of the diets used in this study.

	Concentration (mM)						
	AP3	Leu0	Leu80	Phe0	Phe60	Sugar	Sugar+ Vitamins
**Sugar**							
Sucrose	584,28	584,28	584,28	584,28	584,28	584,28	584,28
**Amino-acid**							
Alanine	20,06	20,06	20,06	20,06	20,06	/	/
β-Alanine	0,70	0,70	0,70	0,70	0,70	/	/
Arginine	14,06	14,06	14,06	14,06	14,06	/	/
Asparagine H20	19,88	19,88	19,88	19,88	19,88	/	/
Aspartic acid	6,63	6,63	6,63	6,63	6,63	/	/
Cystéine	2,44	2,44	2,44	2,44	2,44	/	/
Glutamic acid	10,15	10,15	10,15	10,15	10,15	/	/
Glutamine	30,49	30,49	30,49	30,49	30,49	/	/
Glycine	22,19	22,19	22,19	22,19	22,19	/	/
Histidine HCl H2O	6,49	6,49	6,49	6,49	6,49	/	/
Isoleucine (allo free)	12,56	12,56	12,56	12,56	12,56	/	/
Leucine	17,65	/	80	17,65	17,65	/	/
Lysine HCl	19,22	19,22	19,22	19,22	19,22	/	/
Méthionine	4,85	4,85	4,85	4,85	4,85	/	/
Ornithine HCl	0,56	0,56	0,56	0,56	0,56	/	/
Phénylalanine	17,83	17,83	17,83	/	60	/	/
Proline	11,23	11,23	11,23	11,23	11,23	/	/
Sérine	11,83	11,83	11,83	11,83	11,83	/	/
Thréonine (allo free)	10,67	10,67	10,67	10,67	10,67	/	/
Tryptophan	2,09	2,09	2,09	2,09	2,09	/	/
Tyrosine	2,13	2,13	2,13	2,13	2,13	/	/
Valine	16,29	16,29	16,29	16,29	16,29	/	/
**Vitamins**							
p-aminobenzoic acid	0,73	0,73	0,73	0,73	0,73	/	0,73
L-Ascorbic acid	5,68	5,68	5,68	5,68	5,68	/	5,68
Biotin	0,00	0,00	0,00	0,00	0,00	/	0,00
D-calcium penthothenate	0,10	0,10	0,10	0,10	0,10	/	0,10
Choline chloride	3,58	3,58	3,58	3,58	3,58	/	3,58
Folic acid	0,02	0,02	0,02	0,02	0,02	/	0,02
i-Inositol	2,33	2,33	2,33	2,33	2,33	/	2,33
Nicotinamide	0,82	0,82	0,82	0,82	0,82	/	0,82
Pyridoxin HCl	0,12	0,12	0,12	0,12	0,12	/	0,12
Riboflavin	0,01	0,01	0,01	0,01	0,01	/	0,01
Thiamine HCl	0,07	0,07	0,07	0,07	0,07	/	0,07
**Others**							
$CuSO_4$, 5H20	0,02	0,02	0,02	0,02	0,02	/	/
$FeCl_3$, 6H2O	0,16	0,16	0,16	0,16	0,16	/	/
$MnCl_2$, 4H2O	0,03	0,03	0,03	0,03	0,03	/	/
NaCl	0,43	0,43	0,43	0,43	0,43	/	/
$ZnCl_2$	0,06	0,06	0,06	0,06	0,06	/	/
Calcium citrate	0,18	0,18	0,18	0,18	0,18	/	/
Cholesteryl benzoate	0,05	0,05	0,05	0,05	0,05	/	/
$MgSO_4$, 7H20	9,82	9,82	9,82	9,82	9,82	/	/
KH_2PO_4	18,37	18,37	18,37	18,37	18,37	/	/

Supplementary text

Statistical models, ANOVA tables and post hoc tests are presented in the order in which the figures appear in the paper (p-value are in bold).

1. Cumulated fecundity analysis (Supplementary Figure 2A - Leucine)

Model:

```
gomp <- as.formula("fecundity ~ K * exp (log(N0/K)*exp(-r0*daysc))") # Gompertz model
m2lg <- nlme(gomp, data = fleu.g,
        random = K + N0 + r0 ~ 1, # random effects (3 parameters)
        fixed = list(K ~ diet, N0 ~ diet, r0 ~ diet), # (3 x 3 parameters)
        start=c(29,26, 28, 2.3, 1.7, 1.3, 0.49, 0.48, 0.48),
        control = nlmeControl(maxIter = 1000, msMaxIter = 250, niterEM = 100))
```

ANOVA table:

	Value	Std.Error	DF	t-value	p-value
K.(Intercept)	33.72472	0.5430451	361	62.10298	**0.0000**
K.dietLeu0	-4.61268	0.7596004	361	-6.07250	**0.0000**
K.dietLeu80	-2.12471	0.8837890	361	-2.40409	**0.0167**
N0.(Intercept)	3.22767	0.2367247	361	13.63471	**0.0000**
N0.dietLeu0	-1.35876	0.3162772	361	-4.29611	**0.0000**
N0.dietLeu80	-1.54944	0.3215990	361	-4.81792	**0.0000**
r0.(Intercept)	0.26545	0.0198706	361	13.35910	**0.0000**
r0.dietLeu0	0.00918	0.0288850	361	0.31794	**0.7507**
r0.dietLeu80	0.00480	0.0305148	361	0.15726	**0.8751**

2. Cumulated fecundity analysis (Supplementary Figure 2B - Phenylalanine)

Model:

```
gomp <- as.formula("fecundity ~ K * exp (log(N0/K)*exp(-r0*daysc))") # Gompertz model
m2pg <- nlme(gomp, data = fphe.g,
         random = K + N0 + r0 ~ 1, # random effects (3 parameters)
         fixed = list(K ~ diet, N0 ~ diet, r0 ~ diet), # (3 x3 parameters)
         start=c(33,29, 31, 3.2, 2.2, 2.0, 0.26, 0.27, 0.27),
         control = nlmeControl(maxIter = 1000, msMaxIter = 250, niterEM = 100))
```

ANOVA table:

	Value	Std.Error	DF	t-value	p-value
K.(Intercept)	36.09293	1.6569979	403	21.782125	**0.0000**
K.dietF0	-5.07288	2.2765810	403	-2.228290	**0.0264**
K.dietF60	-2.45465	2.2959917	403	-1.069102	**0.2857**
N0.(Intercept)	1.89515	0.2957643	403	6.407650	**0.0000**
N0.dietF0	-0.30834	0.4064970	403	-0.758525	**0.4486**
N0.dietF60	-0.07479	0.4088980	403	-0.182913	**0.8550**
r0.(Intercept)	0.24073	0.0122872	403	19.591501	**0.0000**
r0.dietF0	0.02486	0.0180108	403	1.380535	**0.1682**
r0.dietF60	0.03527	0.0181117	403	1.947274	**0.0522**

3. Bacteriocyte counting (Figure 2A - Leucine)

Model: lm1 <- lm(bac~stage*diet)

ANOVA table:

	Df	Sum Sq	Mean Sq	F value	Pr(>F)

stage	5	3518.8	703.77	70.1344	**< 2.2e-16** ***
diet	2	542.4	271.21	27.0271	**7.432e-11** ***
stage:diet	10	199.3	19.93	1.9857	**0.03796** *
Residuals	162	1625.6	10.03		

Post hoc tests:

contrast	estimate	SE	df	t.ratio	p.value
stage = J05					
AP3 - 0Leu0	3.6	1.42	162	2.541	**0.03**
AP3 - Leu80	6.7	1.42	162	4.729	**<.0001**
0Leu0 - Leu80	3.1	1.42	162	2.188	**0.0762**
stage = J07					
AP3 - 0Leu0	-0.2	1.42	162	-0.141	**0.9891**
AP3 - Leu80	5.4	1.42	162	3.812	**0.0006**
0Leu0 - Leu80	5.6	1.42	162	3.953	**0.0003**
stage = J09					
AP3 - 0Leu0	-1.3	1.42	162	-0.918	**0.6299**
AP3 - Leu80	2.9	1.42	162	2.047	**0.1044**
0Leu0 - Leu80	4.2	1.42	162	2.965	**0.0097**
stage = J12					
AP3 - 0Leu0	-1.9	1.42	162	-1.341	**0.3745**
AP3 - Leu80	1.7	1.42	162	1.200	**0.4549**
0Leu0 - Leu80	3.6	1.42	162	2.541	**0.0320**
stage = J14					
AP3 - 0Leu0	-2.8	1.42	162	-1.976	**0.1213**
AP3 - Leu80	1.9	1.42	162	1.341	**0.3745**
0Leu0 - Leu80	4.7	1.42	162	3.318	**0.0032**
stage = J16					
AP3 - 0Leu0	-2.2	1.42	162	-1.553	**0.2692**
AP3 - Leu80	0.7	1.42	162	0.494	**0.8742**
0Leu0 - Leu80	2.9	1.42	162	2.047	**0.1044**

P value adjustment: tukey method for comparing a family of 3 estimates

4. Bacteriocyte counting (Figure 2B - Phenylalanine)

Model: lm1 <- lm(bac~stage*diet)

ANOVA table:

	Df	Sum Sq	Mean Sq	F value	Pr(>F)
stage	5	5891.1	1178.23	112.516	**< 2.2e-16** ***
diet	2	1423.6	711.80	67.974	**< 2.2e-16** ***
stage:diet	10	1436.9	143.69	13.722	**< 2.2e-16** ***
Residuals	162	1696.4	10.47		

Post hoc tests:

contrast	estimate	SE	df	t.ratio	p.value
stage = J05					
AP3 - F0	-9.0	1.45	162	-6.219	**<.0001**
AP3 - F60	-4.2	1.45	162	-2.902	**0.0117**
F0 - F60	4.8	1.45	162	3.317	**0.0032**
stage = J07					
AP3 - F0	-4.5	1.45	162	-3.110	**0.0062**
AP3 - F60	-1.4	1.45	162	-0.967	**0.5985**
F0 - F60	3.1	1.45	162	2.142	**0.0847**
stage = J09					

AP3 - F0	-5.1	1.45	162	-3.524	**0.0016**
AP3 - F60	-0.7	1.45	162	-0.484	**0.8791**
F0 - F60	4.4	1.45	162	3.040	**0.0077**
stage = J12					
AP3 - F0	-5.2	1.45	162	-3.593	**0.0013**
AP3 - F60	-2.8	1.45	162	-1.935	**0.1323**
F0 - F60	2.4	1.45	162	1.658	**0.2245**
stage = J14					
AP3 - F0	-7.0	1.45	162	-4.837	**<.0001**
AP3 - F60	-2.4	1.45	162	-1.658	**0.2245**
F0 - F60	4.6	1.45	162	3.179	**0.0050**
stage = J16					
AP3 - F0	-3.4	1.45	162	-2.349	**0.0520**
AP3 - F60	14.5	1.45	162	10.020	**<.0001**
F0 - F60	17.9	1.45	162	12.369	**<.0001**

P value adjustment: tukey method for comparing a family of 3 estimates

5. Bacteriocyte surface analysis (Supplementary Figure 3 – Leucine and Phenylalanine)

Model: sc= stage:condition ; lmcwsc=lme(fixed=surf~condition*stage,random=list(aphid=~1), method="REML", weights = varIdent(form = ~1 | sc), control = nlmeControl(maxIter = 1000, msMaxIter = 250, niterEM = 100))

ANOVA table:

	numDF	denDF	F-value	p-value
(Intercept)	1	3519	4800.734	**<.0001**
condition	3	144	15.751	**<.0001**
stage	5	144	17.612	**<.0001**
condition:stage	15	144	1.031	**0.4275**

Post hoc tests:

contrast	estimate	SE	df	t.ratio	p.value
stage = J05					
AP3 - Leu0	68.7	355	144	0.193	**0.9974**
AP3 - Leu80	598.6	353	144	1.697	**0.3288**
AP3 - Phe0	-456.9	353	144	-1.293	**0.5688**
Leu0 - Leu80	530.0	353	144	1.499	**0.4405**
Leu0 - Phe0	-525.6	354	144	-1.484	**0.4497**
Leu80 - Phe0	-1055.6	352	144	-3.001	**0.0165**
stage = J07					
AP3 - Leu0	-408.5	364	144	-.121	**0.6774**
AP3 - Leu80	190.2	364	144	0.522	**0.9536**
AP3 - Phe0	-792.3	367	144	-2.161	**0.1393**
Leu0 - Leu80	598.7	362	144	1.654	**0.3519**
Leu0 - Phe0	-383.9	364	144	-1.053	**0.7183**
Leu80 - Phe0	-982.5	364	144	-2.698	**0.0386**
stage = J09					
AP3 - Leu0	129.6	358	144	0.362	**0.9837**
AP3 - Leu80	-100.7	361	144	-0.279	**0.9924**
AP3 - Phe0	-1124.6	360	144	-3.121	**0.0116**
Leu0 - Leu80	-230.3	361	144	-0.637	**0.9198**
Leu0 - Phe0	-1254.2	361	144	-3.472	**0.0038**
Leu80 - Phe0	-1023.9	364	144	-2.815	**0.0281**
stage = J12					
AP3 - Leu0	-405.6	360	144	-1.126	**0.6742**

AP3 - Leu80	191.4	360	144	0.532	**0.9511**
AP3 - Phe0	-1287.7	365	144	-3.523	**0.0032**
Leu0 - Leu80	597.0	361	144	1.652	**0.3531**
Leu0 - Phe0	-882.1	367	144	-2.403	**0.0811**
Leu80 - Phe0	-1479.1	367	144	-4.034	**0.0005**
stage = J14					
AP3 - Leu0	-310.0	358	144	-0.867	**0.8219**
AP3 - Leu80	229.3	362	144	0.634	**0.9210**
AP3 - Phe0	-73.0	358	144	-0.204	**0.9970**
Leu0 - Leu80	539.3	360	144	1.497	**0.4422**
Leu0 - Phe0	237.0	356	144	0.665	**0.9101**
Leu80 - Phe0	-302.3	361	144	-0.838	**0.8363**
stage = J16					
AP3 - Leu0	-216.3	362	144	-0.598	**0.9324**
AP3 - Leu80	145.8	359	144	0.406	**0.9773**
AP3 - Phe0	-860.3	361	144	-2.381	**0.0853**
Leu0 - Leu80	362.1	361	144	1.004	**0.7473**
Leu0 - Phe0	-644.0	363	144	-1.776	**0.2893**
Leu80 - Phe0	-1006.1	360	144	-2.792	**0.0300**

P value adjustment: tukey method for comparing a family of 4 estimates

6. Cumulated fecundity analysis (Supplementary Figure 4 - Starvation)

Model:

```
gomp <- as.formula("fecundity ~ K * exp (log(N0/K)*exp(-r0*daysc))") # Gompertz model
m2sg <- nlme(gomp, data <- fstarv.g,
        random <- K + N0 + r0 ~ 1, # random effect (3 parameters)
        fixed <- list(K ~ diet, N0 ~ diet, r0 ~ diet), # 2 x3 parameters
        start<-c(100,100, 5, 5, 0.3, 0.3))
```

ANOVA table:

	Value	Std.Error	DF	t-value	p-value
K.(Intercept)	94.89515	2.796564	450	33.93276	**0.0000**
K.diets24h	-1.53377	4.207814	450	-0.36451	**0.7157**
N0.(Intercept)	7.64278	0.794998	450	9.61359	**0.0000**
N0.diets24h	-3.66505	1.172950	450	-3.12464	**0.0019**
r0.(Intercept)	0.20154	0.008306	450	24.26339	**0.0000**
r0.diets24h	-0.02381	0.012299	450	-1.93573	**0.0535**

7. Aphid weight (Supplementary Figure 8A - Starvation)

Model: lm1 <- lm(w~stage*diet)

ANOVA table:

	Df	Sum Sq	Mean Sq	F value	Pr(>F)
stage	6	158.873	26.4788	152.667	**< 2.2e-16** ***
diet	1	15.358	15.3584	88.551	**2.987e-16** ***
stage:diet	6	11.147	1.8579	10.712	**1.333e-09** ***
Residuals	126	21.854	0.1734		

Post hoc tests:

contrast	estimate	SE	df	t.ratio	p.value
stage = J05					
plante - starvation_24h	0.309	0.186	126	1.659	**0.0996**
stage = J07					
plante - starvation_24h	0.816	0.186	126	4.381	**<.0001**

stage = J09					
plante - starvation_24h	1.966	0.186	126	10.556	**<.0001**
stage = J12					
plante - starvation_24h	0.497	0.186	126	2.668	**0.0086**
stage = J15					
plante - starvation_24h	0.225	0.186	126	1.208	**0.2293**
stage = J19					
plante - starvation_24h	0.549	0.186	126	2.948	**0.0038**
stage = J23					
plante - starvation_24h	0.275	0.186	126	1.477	**0.1423**

8. Bacteriocyte counting (Figure 5A - Starvation)

Model: glm1<- glm(bac ~ stage * diet, family = "quasipoisson")

ANOVA table:

Analysis of Deviance Table (Type II tests)

	LR	Chisq Df	Pr(>Chisq)
stage	329.72	6	**< 2.2e-16** ***
diet	131.71	1	**< 2.2e-16** ***
stage:diet	52.30	6	**1.626e-09** ***

Post hoc tests:

contrast	estimate	SE	df	z.ratio	p.value
stage = J05					
plante - starvation_24h	0.58543	0.0642	Inf	9.120	**<.0001**
stage = J07					
plante - starvation_24h	0.14510	0.0516	Inf	2.810	**0.0049**
stage = J09					
plante - starvation_24h	0.35037	0.0561	Inf	6.249	**<.0001**
stage = J12					
plante - starvation_24h	0.33236	0.0593	Inf	5.603	**<.0001**
stage = J15					
plante - starvation_24h	0.20884	0.0589	Inf	3.546	**0.0004**
stage = J19					
plante - starvation_24h	0.15242	0.0670	Inf	2.275	**0.0229**
stage = J23					
plante - starvation_24h	-0.00519	0.0720	Inf	-0.072	**0.9425**

Results are given on the log (not the response) scale.

9. Bacteriocyte surface analysis (Figure 5B - Starvation)

Model: lmcws <- lme(fixed=surf ~ condition * stage, random=list(aphid=~1), method="REML",
weights = varIdent(form = ~1 | stage))

ANOVA table:

	numDF	denDF	F-value	p-value
(Intercept)	1	3709	3578.898	**<.0001**
condition	1	126	4.381	**0.0383**
stage	6	126	30.029	**<.0001**
condition:stage	6	126	6.234	**<.0001**

Post hoc tests:

contrast	estimate	SE	df	t.ratio	p.value
stage = J05					

plante - s24	1486.237	710	126	2.093	**0.0383**
stage = J07					
plante - s24	2440.966	718	126	3.400	**0.0009**
stage = J09					
plante - s24	2737.990	731	126	3.747	**0.0003**
stage = J12					
plante - s24	-0.884	726	126	-0.001	**0.9990**
stage = J15					
plante - s24	-495.003	721	126	-0.686	**0.4939**
stage = J19					
plante - s24	241.047	725	126	0.333	**0.7400**
stage = J23					
plante - s24	-2459.543	732	126	-3.362	**0.0010**

10. Symbionts counting (Figure 5C - Starvation)

Model: glm1 <- glm(count~stage*diet, family = "quasipoisson")

ANOVA table:

	LR	Chisq Df	Pr(>Chisq)
stage	1399.68	6	**< 2.2e-16** ***
diet	19.98	1	**7.814e-06** ***
stage:diet	56.88	6	**1.933e-10** ***

Post hoc tests:

contrast	estimate	SE	df	z.ratio	p.value
stage = J05					
plante - s24	1.3107	0.5633	Inf	2.327	**0.0200**
stage = J07					
plante - s24	1.2025	0.3364	Inf	3.575	**0.0004**
stage = J09					
plante - s24	0.9216	0.1602	Inf	5.754	**<.0001**
stage = J12					
plante - s24	0.2886	0.0896	Inf	3.220	**0.0013**
stage = J15					
plante - s24	-0.0957	0.0776	Inf	-1.233	**0.2176**
stage = J19					
plante - s24	0.0270	0.0872	Inf	0.310	**0.7569**
stage = J23					
plante - s24	0.2706	0.1089	Inf	2.486	**0.0129**
